# How to Apply Positive-Reinforcement-Based Training for Self-Loading and Self-Unloading in Dromedary Camels

**DOI:** 10.3390/ani16071103

**Published:** 2026-04-03

**Authors:** Naod Thomas Masebo, Asim Faraz, Maria Gaia Angeloni, Faizan Saleem, Hassan Qadir Buzdar, Barbara Padalino

**Affiliations:** 1 Department of Agricultural and Food Sciences, University of Bologna, 40127 Bologna, Italy; naodthomas.masebo2@unibo.it (N.T.M.); mariagaia.angeloni@unibo.it (M.G.A.); 2 Department of Livestock and Poultry Production, Bahauddin Zakariya University, Multan 60800, Pakistan; drasimfaraz@bzu.edu.pk (A.F.); mrfs2632@gmail.com (F.S.); hassanbuzdar47@gmail.com (H.Q.B.); 3 Faculty of Science and Engineering, Southern Cross University, Lismore, NSW 2480, Australia

**Keywords:** behavior, clicker training, handling, infrared thermography (IRT), transport, welfare

## Abstract

This study aimed to describe how to train dromedary camels to self-load and unload using positive-reinforcement-based training and to examine its effects on behavior and welfare. Twelve camels, six unbroken male camels (group A) and six broken mixed-gender camels (group B), underwent nine days of positive reinforcement training, after an initial day of behavioral tests. The training session included six phases: loading the clicker, approaching the truck, ramp, truck, unloading, and returning to the station. Eye temperature was measured before and after training using infrared thermography, and training was recorded for further behavioral analysis. Overall, eight camels (66.7%) loaded and unloaded successfully at least once. The average total and daily training duration were 72 and 8.5 min per camel, respectively, with the fastest camel able to load taking only 30 min of total training over five days. Loading the clicker phase decreased by 41% per additional training day (*p* < 0.001) and was higher in camels that completed the training session compared to unsuccessful individuals (*p* = 0.027). Similarly, the time required to approach the truck decreased significantly across training days (*p* < 0.001). Training day had no effect on the duration of the ramp phase; however, this phase was overall shorter in the group of successful camels (*p* = 0.038). Loading inside the truck increased by 50% with each additional training day (*p* = 0.007). Camels in group B had significantly lower maximum eye temperatures than those in group A (*p* = 0.019), with no significant effect of day or time (*p* = 0.373). In conclusion, our study shows that training dromedary camels to self-load and unload is possible and does not increase eye temperature. It could mitigate transport stress, improving the safety of handlers and camels.

## Simple Summary

Transporting farm animals is potentially hazardous and exposes the animals to negative welfare consequences. Loading and unloading are considered the two most stressful phases of transport. This study describes how to train a dromedary camel to self-load and unload using positive-reinforcement-based training. Twelve camels found in two different locations, six male camels (group A) and six camels of mixed gender (group B), underwent nine days of positive reinforcement training after an initial day of behavioral tests. Eye temperature was measured before and after training using infrared thermography, and training was recorded for further behavioral analysis. Camels successfully learned to self-load and unload in a short period of time without signs of fear and distress. Overall, eight camels loaded and unloaded successfully at least once. The average total and daily training duration were 72 and 8.5 min per camel, respectively, with the fastest camel able to load taking only 30 min of total training over five days. Camels in group B had significantly lower maximum eye temperatures than those in group A, with no significant effect of day or time. In conclusion, this training showed that dromedary camels can be trained to self-load and unload; hence, the training could minimize the negative welfare impact of transport and injuries to both camels and handlers.

## 1. Introduction

Transporting farm animals is a potentially stressful and risky practice that can negatively affect their welfare, regardless of whether transport occurs by road, rail, air, or sea [1,2]. The impact of transportation on animal welfare can be minimized by applying suitable and best practices [2,3]. Farm animals are transported to various locations for different purposes, such as to other farms, collection centers, and slaughterhouses [4,5]. Dromedary camels are no different, and are transported to markets, slaughterhouses, racing tracks (sport camels) and other locations commonly by vehicles or by foot and are exported to other countries also via sea using vessels [6]. Transportation exposes them to various welfare consequences and physiological stress during preparation or collection, loading into the vehicle, during the journey, and unloading [6,7]. Loading and unloading are considered the two most stressful phases of transport [6,8,9,10] and are also dangerous for handlers [11]. In addition to other factors influencing loading and unloading, animal factors, such as previous experience, handling, social behavior, and health, also influence these procedures [12]. In most camel-rearing countries, the loading and unloading of camels at markets and slaughterhouses are traditional [13]. There is excessive use of force, pulling by the nose or tail, violent shouting by the handlers, and striking with a stick [13]. Usually, the camels are tied and shackled and carried by a human or crane to a vehicle [13].

Training animals to self-load and unload using positive reinforcement methods has been suggested as an effective practice to reduce transport stress [11,14,15]. Positive reinforcement is a consequence that increases the likelihood of behaviors recurring if it is presented at an appropriate time [16,17]. Positive reinforcement can be primary, such as food, and secondary, such as the ‘click’ of a clicker [16]. Training animals to self-load using positive reinforcement has been applied in horses and calves [9,11,14]. Therefore, this study hypothesized that camels would learn to self-load and unload from a stationary vehicle using positive reinforcement training without showing any evident signs of distress and fear in a short period of time.

Infrared thermography (IRT) is widely utilized as a non-invasive method for assessing stress in various livestock species [18,19,20,21]. The eye temperature measured using IRT is an effective indicator of whether animals are experiencing stress, fear, or pain following training and other husbandry practices [9,19,21,22]. This study aimed to describe how to train a dromedary to self-load and unload using positive-reinforcement-based training and its effects on behavior and welfare using IRT.

## 2. Materials and Methods

### 2.1. Animals

**Figure 1:**
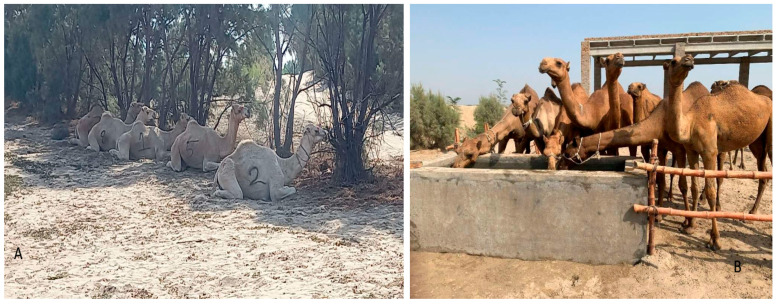
Camels used in the study ((**A**) group A camels resting under trees; (**B**) group B camels at the drinking point).

The study was conducted in a commercial mixed farm (crop and livestock) from 3 to 14 October 2025. Although the farm houses other livestock species (sheep, cattle, and buffalo), dromedary camels were introduced recently. The study included 20 camels (*Camelus dromedarius*) belonging to the Kohi and Barilya breeds, which were kept for meat and milk purposes, respectively. These animals had been newly introduced to the farm. The camels were kept in two locations on the same farm, group A and group B. Group A was composed of eight males, recently purchased and brought to the farm to establish a new camel herd, and were kept in an open area under trees while the shade was under construction (Figure 1A). They were unbroken camels with minimal experience in human interaction and handling. Group B was composed of 12 camels (eight females, three males, and one male calf) and was kept in a pen made of bamboo and brick poles with precast concrete slab roofs. They were broken camels, had been kept at the farm longer, and had experience in human handling. The pen they were kept in had an artificial shelter where the camels could protect themselves from the sun and stay inside whenever needed. The pen also had watering and feeding troughs. Water and feed were offered ad libitum only in group B (Figure 1B). Camels of both groups were used to browsing during the day and they were housed and fed Rhodes grass hay at night. During the study period, they were also fed with a concentrate used as a reward to make them more familiar with it. The senior authors initially selected and marked all the camels in group A and then randomly selected 7/12 from group B. Then, the fifteen selected camels were health checked, and three animals were excluded, two for respiratory problems in group A (ID 1, 8) and one for dental and dermatological problems, which was under treatment, in group B (ID 13). After that, the 12 remaining camels (7 males and 5 females; 6 for each group) were considered fit for the training. They had an average age of 4.4 years and were white or brown in color. All males were sexually mature, while among the females, three were pregnant and two were non-pregnant (Supplementary Table S1).

### 2.2. The Training Protocol

**Figure 2:**
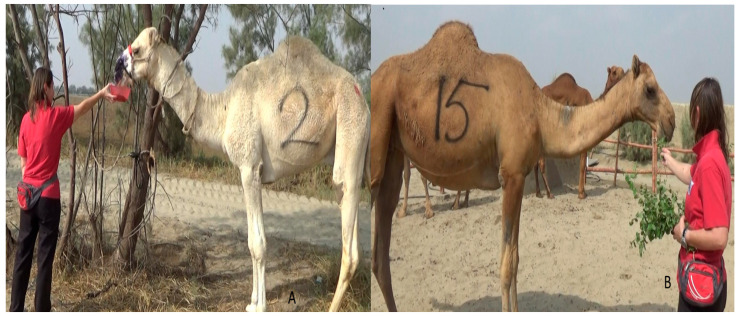
Showing the trainer performing a food preference test ((**A**) a camel accepting concentrate in group A; (**B**) a camel accepting plants in group B).

The camels underwent positive reinforcement training; in particular, a clicker-training/target-training method was applied [15,17,23]. A feed reward (concentrate) or plant (*Ziziphus mauritiana*)) was used as a primary reinforcer, while the clicker was used as a secondary reinforcer [23]. The nutritional composition of the concentrate used in the training is reported in Supplementary Table S2. The camels were offered food as a reward, and the food was paired with the clicker; then, the clicker was pressed when the camels performed the desired activity. In the first group (A), the trainer used a rope loosely tied around the neck to guide the camels without applying pressure. In the second group (B), no rope was used during training. The camels were trained individually. The duration of each training phase was recorded. The training sessions were conducted between 7:30 a.m. and 11 a.m. All the training sessions were performed by the same trainer (B.P.). During each training session, the trainer stood in front of the camels with training materials (a red bowl used as a target and food reward) at a distance of approximately 0.5 m, and only the trainer gave the reward and commands (e.g., touch the target, follow, and stop). The full protocol lasted 10 days. On the first day, three behavioral tests, namely an approaching test, a modified broken/unbroken test (BUT) [24], and a feed preference test, were conducted. The food preference test was conducted to identify the preferred feed type and, consequently, to use it as a reward during the positive reinforcement training for each camel. The trainer offered either the concentrate (Supplementary Table S2) or green plant (*Ziziphus mauritiana*) to each camel (Figure 2), and individual food preferences were recorded. Based on the outcomes of the behavioral tests, some descriptors of personality were attributed to each camel. Namely, the camels were defined as aggressive when the camel exhibited threatening or harmful actions towards an approaching person, such as threat displays, rearing up, kicking, pushing, and biting [25]; curious when the camel showed interest in exploring something new and showed actions such as making the ears forward, alert, sniffing, or gently touching the hand of a person approaching [26]; friendly when the camel willingly sought or accepted interaction, such as approaching humans voluntarily and standing close without tension [27]; shy when the camel was hesitant and cautious about interaction, moved away slowly when approached, and kept its head slightly turned aside [25]; calm when the camel was relaxed and not reacting strongly to a person approaching or to its surroundings and showing actions such as a lowered head and relaxed neck and minimal movement [25]; uneasy when the camel raised its head and, with a tense neck, it was mildly stressed or uncertain [25]; and fearful when the camel refused to move forward or it moved away from an approaching person [25,28].

On the second day, the training commenced, and the animals were taught to follow the trainer. On the third day, a truck (5.23 × 2.10 m, Mazda brand) was introduced, and the animals were led up to the truck without a ramp and were allowed to feed from the truck’s cargo bed. On the fourth day, a handmade wood ramp (2.20 × 3.5 m) was fitted to the truck, and the animals were trained to self-load and self-unload until the 10th day. The training session was considered successful if the camel put at least one leg on the truck bed, and the training was considered completed when a camel was able to self-load and self-unload for three days in a row. The progress of each camel was considered at the end of each day, and the training was individually adapted and recorded.

The training session was subdivided into up to six phases: loading the clicker, approaching the truck, ramp phase, truck phase, unloading, and going to the station.

#### 2.2.1. Loading the Clicker Phase

**Figure 3:**
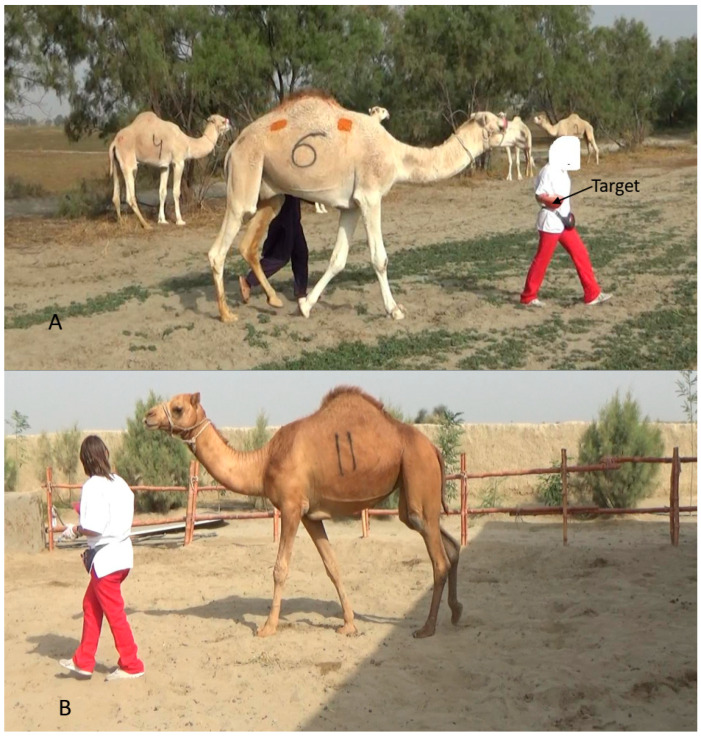
The camels following the trainer, who is holding the target in her hand ((**A**) camel of group A; (**B**) camel of group B).

During this phase, the clicker was loaded, pairing the sound of the clicker with the preferred feed. The trainer presented a target (i.e., a red plastic bowl) in front of each camel, and when the camel touched it, the clicker was pressed, and the camel was rewarded with food. The clicker thus served as a conditioned reinforcer [15]. This procedure was repeated while the trainer guided the camels to follow her to different locations within the training field and then back to their original station (Figure 3).

#### 2.2.2. Approaching the Truck Phase

**Figure 4:**
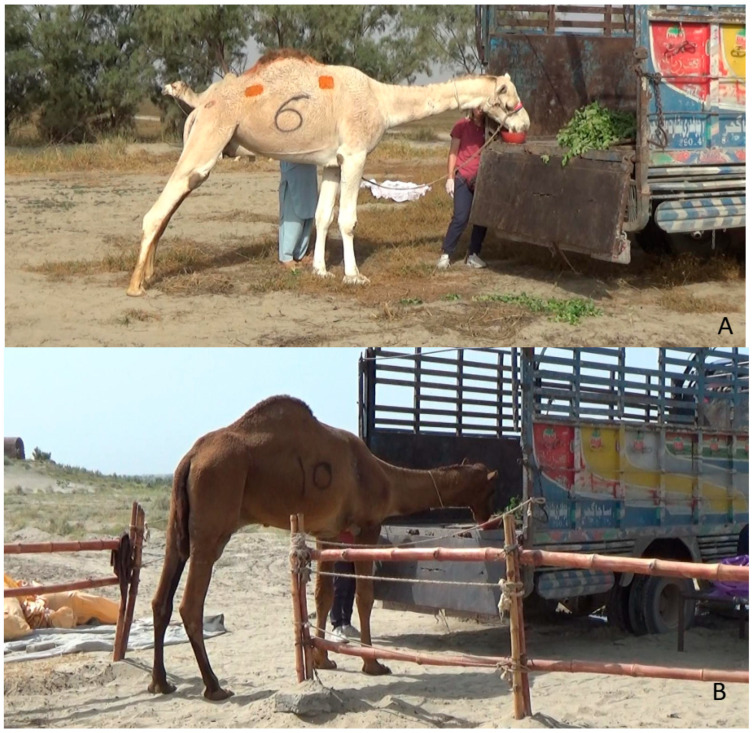
Camels eating from the cargo bed after a successful approach ((**A**) camel of group A; (**B**) camel of group B).

The camels were guided to approach the truck by following the target kept by the trainer. When a camel moved forward toward the truck and touched the target, the clicker was pressed, and the camel was rewarded with food. This process was repeated until the camels approached the truck. The camels were then allowed to eat from the cargo bed while remaining standing in the field to familiarize and create a positive association with the truck (Figure 4).

#### 2.2.3. Ramp Phase

**Figure 5:**
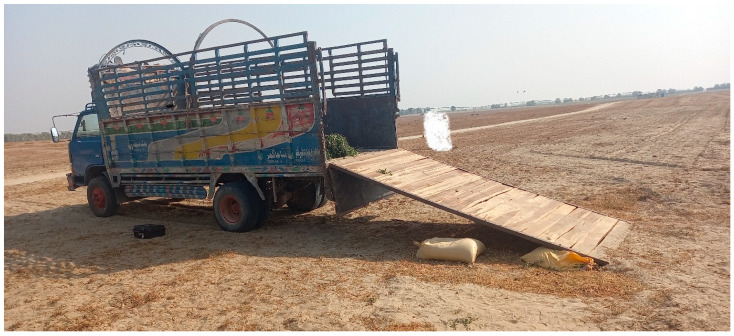
A truck fitted with a handmade modified ramp was used for the training. Since no special vehicle designed to transport camels was found in the study area, a normal truck was fitted with a handmade ramp.

**Figure 6:**
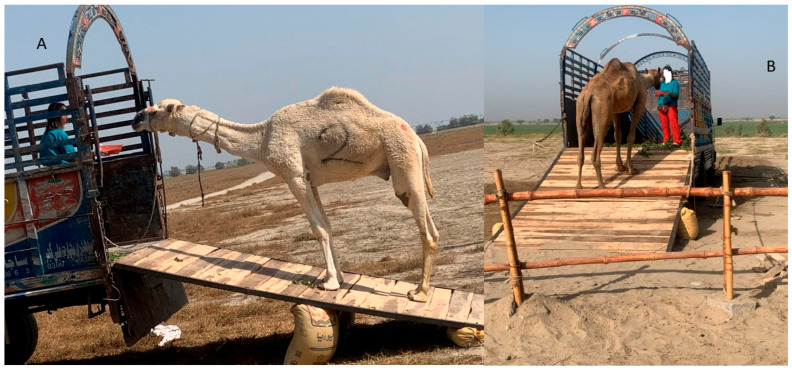
Camels stepping onto the ramp, learning to self-load following the target ((**A**) camel of group A; (**B**) camel of group B).

On the fourth day, the camels were led to approach the ramp (Figure 5) following the trainer. Using the same method, the camels were requested to place a foot onto the ramp and make a step on it to learn to self-load following the target. To facilitate the learning process, the trainer shaped the behavior, asking the camel to move each leg at a time until the camel took a step on the ramp and then walked following the target while self-loading. At the beginning, whenever the camel displayed the desired behavior, a step forward on the ramp, the clicker was pressed, and the camel was rewarded with food. Figure 6 shows camels stepping onto the ramp, learning to self-load.

#### 2.2.4. Truck Phase

**Figure 7:**
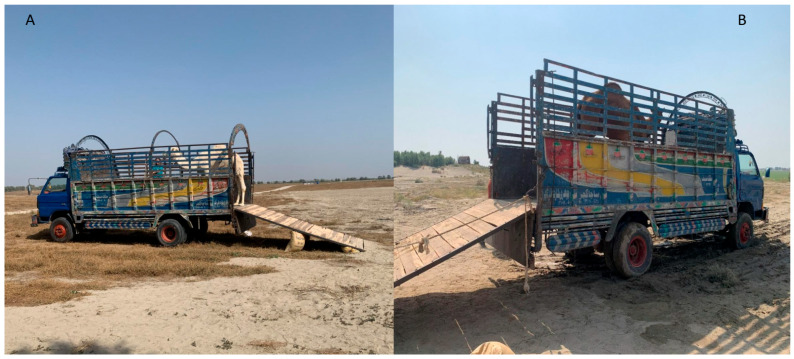
Successfully loaded camels ((**A**) camel of group A; (**B**) camel of group B).

The truck phase started as soon as the camel placed at least one foot on the truck bed. If the camel was in the truck with its full body, it was allowed to remain inside the truck and eat (Figure 7), to create a positive association with the truck.

#### 2.2.5. Unloading Phase

**Figure 8:**
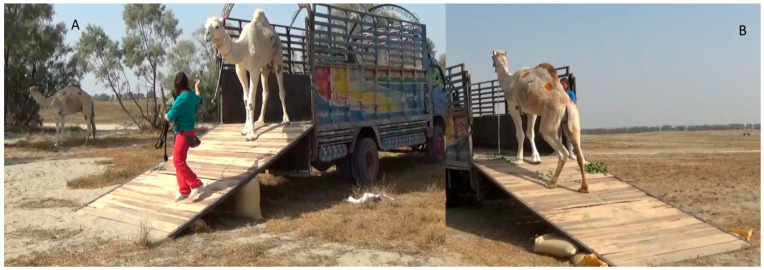
A camel unloading following the trainer using positive reinforcement ((**A**) camel unloading walking forward; (**B**) camel unloading backward).

After reaching the truck, the trainer taught the camel to unload itself, either by backing out or by turning around inside the truck (Figure 8). Using the same method, the clicker was pressed, and the camel was rewarded with food whenever it performed the desired behavior (i.e., stepping down the ramp).

#### 2.2.6. Going Back to the Station

The trainer led the camel back to their location using the same methods of positive reinforcement.

### 2.3. Behavioral Evaluation

For each camel, the training duration of each day was video-recorded using a digital Sony HDR-CX405 camera (Sony Inc., Minato, Tokyo, Japan). The video recordings of the training sessions were analyzed, starting from the second day of training, to assess and understand the camels’ progress in the loading procedure. The ethogram used to analyze the behavior of dromedary camels is presented in Table 1. The behavior of camels during the training was analyzed with a focal animal sampling continuous recording method [29] using the software Solomon Coder (version: beta 17.03.22). The duration and frequency of each behavior were recorded. The behavioral states, such as following, standing, feeding, eating from the ramp, and eating from the truck, were recorded by evaluating their duration (seconds), while behavioral events (e.g., steps forward on the ramp, alert behavior, and avoidance behaviors) were recorded considering their frequency (n). In addition, the latency to the first step on the ramp was calculated as the elapsed time (in seconds) from the start of the ramp phase (i.e., the camel stopped in front of the ramp) until the camel placed its first leg on the ramp.

### 2.4. Thermography of the Eye

Infrared thermography (IRT) images of either the left or the right eye of the camels involved in the training were captured before training and immediately after training using an infrared thermal camera (FLIR E76 24; FLIR Systems AB, Danderyd, Sweden). Before each use, the camera was calibrated using the environmental temperature and relative humidity recorded by a weather station (Kestrel 4000, Wet Des Moines, IA, USA). All images were captured at the same angle (90°) at a distance of 1 m. The pictures were analyzed using software (FLIR Tools^®^ 5.X) to extract the caruncle temperature, measuring the maximum temperature (°C) within a circular area drawn around the caruncle.

### 2.5. Statistical Analysis

All data were analyzed using descriptive statistics. Stratified descriptive statistics by camel ID, training success (i.e., whether the camel successfully completed at least one training session or not), and day (i.e., day of training) were also performed. Continuous variables are reported as mean ± standard deviation (SD), minimum (min), and maximum (max), while categorical variables are reported as counts and percentages. Descriptive statistics were performed using SPSS^®^ 25 statistical software package (SPSS^®^ Inc, Chicago, IL, USA).

#### 2.5.1. Behavioral Data

Regression analyses were conducted to evaluate the effect of the day, training success, and their interaction on the behavioral data recorded during the training sessions. When the interaction term was not statistically significant, it was removed, preferring a more parsimonious model with better fit to the data.

Behavioral events were analyzed using separate negative binomial generalized linear mixed models (GLMMs) to manage count-type response variables that showed evidence of overdispersion. Training duration was included as an offset to account for variation in exposure time due to differences in training length across sessions. Behavioral events with a median equal to zero were excluded from regression analyses. For the latency time to the first step on the ramp, a linear mixed-effects model (LMM) was fitted on the log-transformed outcome variable. Log-transformation was used here to normalize the residual distribution, reduce skewness, and stabilize variance, thus improving the overall model fit. Behavioral states were normalized by dividing their duration by total training duration and analyzed using beta generalized linear mixed model (GLMM). When values at the upper boundary occurred (i.e., in a few cases where camels spent the entire training session feeding), the transformation proposed by Smithson and Verkuilen [30] was applied to accommodate boundary values.

Finally, the duration of the individual training phases was analyzed using separate LMMs fitted on log-transformed outcome variables. For phases in which a substantial proportion of zero values was observed, a two-step modeling approach was employed. First, a binomial GLMM was fitted to the full dataset to assess the probability of the phase’s occurrence (i.e., whether the behavior was performed at all). Second, an LMM was fitted on the subset of positive values to investigate the duration of the phase when it was actually performed. This approach was applied to the ‘loading the clicker’ and ‘truck’ phases. For the ‘truck’ and ‘unloading’ phases, only the effect of training day was included in the model, as these phases could, by definition, be performed only by camels that completed the training successfully. The training phase ‘going back to the station’ was not subjected to regression analysis. Camel ID was included as a random effect in all models tested to account for repeated measures on the same subject.

#### 2.5.2. Training Duration Data

The effect of training day on the duration of training sessions was assessed through a model selection process, comparing linear and quadratic models to identify the best-fitting representation of the observed trend over time. Camel ID was initially included as a random factor; however, given the exploratory aim of this analysis and the absence of detectable inter-individual variability (random-effect variance ≈ 0), model comparisons were conducted using fixed-effects models. As the two models were nested, the comparison was conducted using ANOVA. Model performance was further evaluated by comparing the Akaike Information Criterion (AIC), the Bayesian Information Criterion (BIC), and residual standard error and visually through plots. For the quadratic model, the peak day was also calculated.

#### 2.5.3. Infrared Thermography (IRT) Data

Due to the limited availability of data, the effect of day, time (i.e., before or after training), and group (i.e., A or B) on the maximum eye temperature was analyzed using separate univariable LMMs, with camel ID included as a random effect.

All regression analyses were conducted in the R environment (R Version 4.4.3; www.r-project.org; accessed on 10 January 2026). Results are reported with 95% confidence intervals (CIs) and *p*-values, as estimates ± standard error (SE) for negative binomial, beta, and linear regression models and as odds ratios (OR) for binomial GLMMs. Overall model significance was assessed using likelihood ratio tests (LRTs), and model performance was evaluated using the AIC. Observations with missing values were automatically excluded from the analysis by the software. Significance was set at *p*-values ≤ 0.05, and trends toward significance were set at 0.05 < *p*-values ≤ 0.10.

## 3. Results

### 3.1. Outcomes of the Behavioral Test on the First Day

Table 2 shows the outcomes of the behavioral tests (i.e., temperament, broken/unbroken and approaching test, and food preference). Overall, most of the camels exhibited a friendly, curious, and calm temperament, but some were shy or fearful. Only one camel exhibited an aggressive temperament. Three camels showed negative responses in the approach test, while the others were positive or neutral. All the camels except one accepted food/reward on the first day.

### 3.2. Training Progression and Summary of the Training Outcomes

All camels learned to associate and accept the clicker with reinforcement and to follow the trainer (Supplementary Table S3).

**Figure 9:**
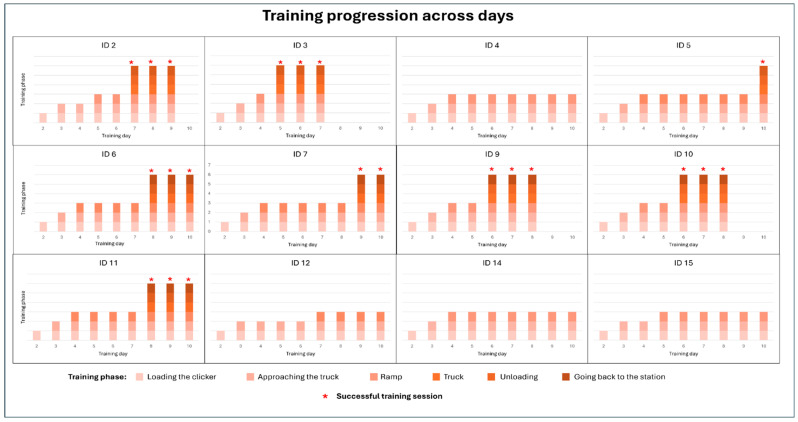
Training progression across days for each camel. Bars represent the different training phases (loading the clicker, approaching the truck, ramp, truck, unloading, and going back to the station). Red asterisks (*) indicate successful training sessions. Three successful sessions in a row were considered as completed training; therefore, camels were no longer trained on the following days.

Camel ID 2 successfully loaded for the first time on day 7, and it completed the training on day 9 after three consecutive successful loading and unloading events. Camel ID 3 was loaded for the first time on day 5, and it completed the training on day 7. Camel ID 5 started climbing the ramp on day 4 but was successfully loaded and unloaded only once on day 10. Camel ID 6 achieved its first load on day 8 and completed the training on day 10. Camel ID 7 climbed the ramp for the first time on day 4 but successfully achieved loading on day 9, and it completed only two consecutive successful sessions. Camel IDs 9 and 10 were loaded for the first time on day 6. They both completed the training successfully on day 8. Camel ID 10 was the fastest camel to load into the truck and took 30 min (1812 s) of training duration. Camel ID 11 was loaded onto the truck for the first time on day 8. It was highly responsive throughout the training period, consistently approaching the truck and successfully climbing the ramp. However, camel IDs 4, 12, 14, and 15 did not load successfully and only walked on the ramp. Camel IDs 4 and 14 remained on the ramp and consumed food placed there, whereas ID 12 extended her neck into the truck to feed from inside. ID 15 consistently showed low motivation to climb the ramp and exhibited increased fearfulness when trained individually. Similarly, ID 12 was generally fearful and difficult to train alone, but her performance improved when training sessions were conducted in the presence of other camels. Figure 9 and Supplementary Table S4 show the detailed day-by-day training progression for each camel, while summaries of the training outcomes, maximum distance achieved on the ramp, time to first successful loading, total training duration, and days of first loading are presented in Table 3.

Overall, eight camels were successfully loaded during the training period, of which six (IDs 2, 3, 6, 9, 10, and 11) successfully completed the training by loading and unloading into the truck three consecutive times. Four camels walked on the ramp but did not complete loading. Despite this, higher percentages of camels successfully reaching the truck were observed as training progressed, with the highest values recorded on days 8 (45.5%), 9 (44.4%), and 10 (37.5%) (Supplementary Table S5). All pregnant camels included in the training (IDs 12, 14, and 15) did not successfully complete the training, whereas the two non-pregnant female camels (IDs 9 and 11) completed the training successfully. Almost all male camels (IDs 2, 3, 5, 6, 7, and 10) successfully completed the training, except for ID 4. Notably, none of the camels regressed during the training: day by day, all individuals either maintained their progress or advanced to subsequent phases but never returned to earlier stages.

The average total (i.e., across the 9 days) training duration was 72 min (4325 s), with an average daily training time of 8.5 min (514 s) per camel. As the training progressed, the observed proportion of camels placing at least one leg on the ramp, walking onto the ramp, and eating from the ramp increased (Supplementary Table S5). All camels involved in the training attempted to walk onto the ramp, with a mean attempt of 1.9 (±1.3) times, while the maximum attempt recorded was 7. The mean distance walked by each camel during the full period of training, the distance the camels climbed onto the ramp, and the number of attempts the camels made to walk up the ramp are fully reported in Supplementary Table S6.

### 3.3. Behavioral Analysis

#### 3.3.1. Descriptive Statistics

**Figure 10:**
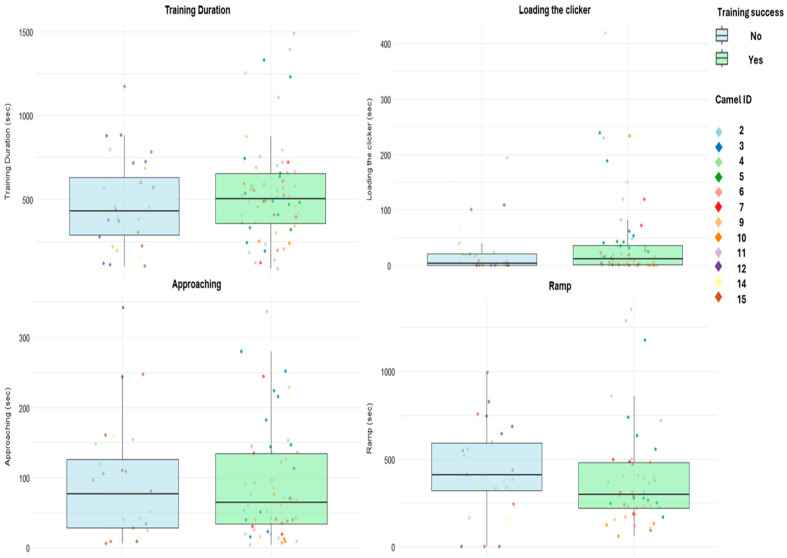
Boxplots representing training phases and overall training duration (seconds) stratified by training success (Yes/No). Boxes represent the interquartile range: the central line indicates the median, and whiskers extend to the most extreme values within 1.5 times the interquartile range. Individual dots represent single observations, with colors corresponding to different camel IDs.

Overall, the ramp phase required more time on average than the other phases, followed by the approaching phase (Table 4), and the overall observed average following durations were slightly higher in the group of successful camels (Supplementary Figure S1). Camels generally needed relatively little time to unload, although with substantial variation among individuals (Table 4 and Supplementary Table S7). Unloading was first performed on the fifth day of training, with the longest observed average durations recorded on days 5 and 6 and the shortest on day 10. Regarding the time spent on the truck, the highest observed average occupancy time was recorded on days 9 and 10 (Table 4). Average training phase durations were generally similar between successful and unsuccessful camels; however, the ramp phase appeared to require longer training in the unsuccessful group (Figure 10). Overall, the observed average training duration was the lowest on day 2 and peaked on day 5 (Table 4).

**Figure 11:**
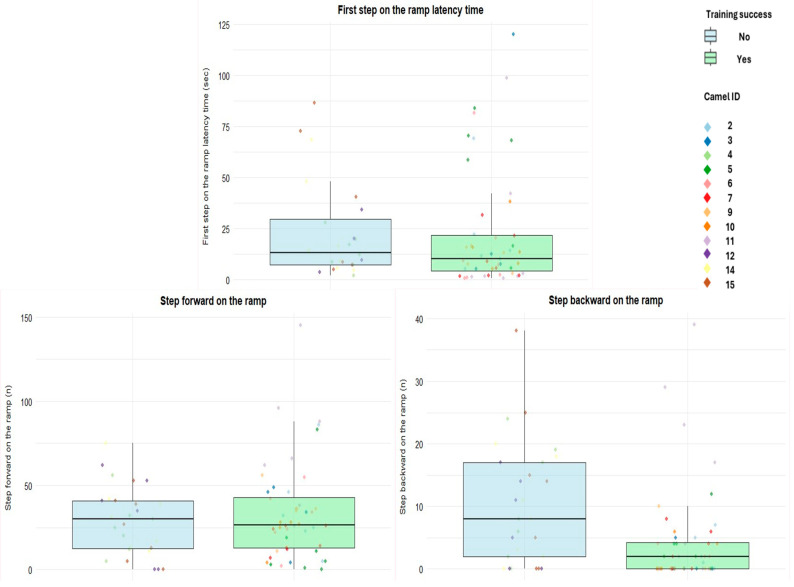
The boxplots represent camels’ movements (steps) on the ramp stratified by training success (Yes/No). Boxes represent the interquartile range: the central line indicates the median, and whiskers extend to the most extreme values within 1.5 times the interquartile range. Individual dots represent single observations, with colors corresponding to different camel IDs.

The observed average number of forward steps on the ramp was lowest on day 4 and reached a peak on day 9. A similar pattern was observed for the forward steps inside the truck (Table 5). The observed number of forward steps on the ramp and the latency to the first step on the ramp were similar between successful and unsuccessful camels, although successful individuals seemed to place the first foot on the ramp after a slightly shorter average time following the stop in front of it (Figure 11). The observed average number of backward steps on the ramp appeared lower in camels that successfully reached the truck at least once, whereas unsuccessful camels more frequently retreated while on the ramp (Figure 11). The observed frequencies of the other behavioral events were generally similar between groups, with successful camels showing a higher average occurrence of sniffing behaviors toward the ramp or truck (Supplementary Figure S2).

Descriptive statistics for duration of training phases, behavioral states, and behavioral events, including camel movements on the ramp and/or truck stratified by camel ID, are summarized in Supplementary Tables S7–S10. Full descriptive statistics of behavioral states and events stratified by training days are reported in Supplementary Tables S11 and S12.

#### 3.3.2. Behavioral Events

The number of steps forward on the ramp increased over training days (estimate = 0.26 ± 0.03, *p* < 0.001), indicating that camels performed more forward steps as training progressed. Conversely, the latency to the first step on the ramp decreased across days (estimate = −0.28 ± 0.07, *p* < 0.001), reflecting a faster start of movement over time. Steps backward on the ramp showed a general increase across days (estimate = 0.56 ± 0.16, *p* < 0.001); however, this increase was smaller in camels that successfully completed the training compared to unsuccessful ones (interaction estimate = −0.46 ± 0.20, *p* = 0.018).

The number of clickers needed tended to decrease slightly across training days (estimate = −0.02 ± 0.01, *p* = 0.098) and was higher in successful camels (estimate = 0.20 ± 0.09, *p* = 0.020). Similarly, the number of rewards required gradually decreased (estimate = −0.06 ± 0.01, *p* < 0.001). Avoidance behavior did not change significantly overall across training days but showed a decreasing trend in successful camels (interaction estimate = −0.18 ± 0.10, *p* = 0.066). Finally, sniffing of the ramp/truck increased across training days (estimate = 0.18 ± 0.08, *p* = 0.032). Model LRT *p*-value, AIC, and full model results for the latency time to the first step on the ramp and all behavioral events analyzed are reported in Supplementary Table S13.

#### 3.3.3. Behavioral States

The percentage of time spent feeding decreased across training days (estimate = −0.31 ± 0.05, *p* < 0.001) and was overall lower in camels that successfully completed the training compared to unsuccessful individuals (estimate = −1.16 ± 0.45, *p* = 0.009). In addition, the decline in feeding behavior across days was less pronounced in successful camels, as indicated by the significant interaction between day and training success (interaction estimate = 0.13 ± 0.06, *p* = 0.033). Regarding standing and following the trainer, the percentage of time spent performing these behaviors, respectively, increased (estimate = 0.26 ± 0.02) and decreased (estimate = −0.26 ± 0.02) across training days (both *p* < 0.001). Model LRT *p*-value, AIC, and full model results for all the behavioral states analyzed are reported in Supplementary Table S14.

#### 3.3.4. Phases of the Training and Overall Training Duration

The odds of observing camels performing the loading clicker phase decreased by approximately 41% for each additional training day (*p* < 0.001; OR = 0.59, 95% CI: 0.46–0.76) and were overall higher in camels that successfully completed the training session almost once compared to unsuccessful individuals (*p* = 0.027; OR = 3.38, 95% CI: 1.15–9.98). When considering only training sessions in which this phase was actually performed, its duration significantly decreased over training days (estimate = −0.35 ± 0.06, *p* < 0.001). Similarly, the time required to approach the truck showed a significant reduction across training days (estimate = −0.34 ± 0.03, *p* < 0.001). Concerning the ramp phase, no significant effect of training day on duration was detected; however, this phase was overall shorter in the group of successful camels (estimate = −0.37 ± 0.18, *p* = 0.038). The odds of loading inside the truck increased by approximately 50% with each additional training day (OR = 1.50, 95% CI: 1.12–2.03, *p* = 0.007), whereas no significant trend in the amount of time spent inside the truck was observed when considering only camels that successfully entered the truck. Finally, the duration of the unloading phase, when successfully performed, significantly decreased over training days (estimate = −0.38 ± 0.12, *p* = 0.001). Supplementary Table S15 reports the model LRT *p*-value, AIC, and full model results for all the analyzed training phases.

**Figure 12:**
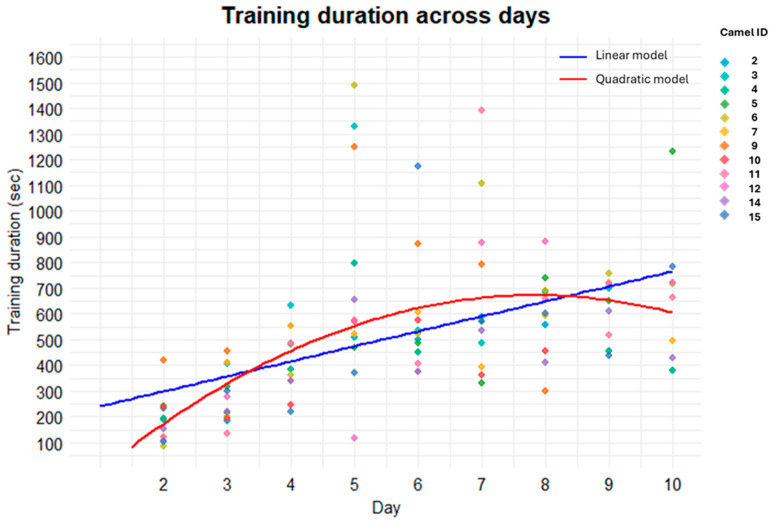
Scatterplot of training duration across training days. Individual points represent single observations, with colors corresponding to different camel IDs. Solid lines represent fitted trends obtained from the model selection process: the blue line indicates the linear model, while the red line represents the quadratic model describing changes in training duration across days.

When considering the duration of the whole training session, the model selection process indicated that the quadratic model provided a significantly better fit to the data than the linear model (*p* < 0.001), suggesting a non-linear trend in training duration across days. Consistently, both AIC and BIC, as well as the residual standard error, were lower for the quadratic model (AIC = 1369.7; BIC = 1380.1; residual standard error = 238.4) compared to the linear one (AIC = 1379.5; BIC = 1387.3; residual standard error = 251.7), further supporting what was stated above. Figure 12 illustrates the observed data and the fitted trends derived from the two alternative models, confirming the non-linear pattern in training duration across training days. According to the quadratic model, training duration increased during the initial phase of the training period, reached a peak around day 8, and subsequently declined.

### 3.4. Infrared Thermography of the Eye

**Figure 13:**
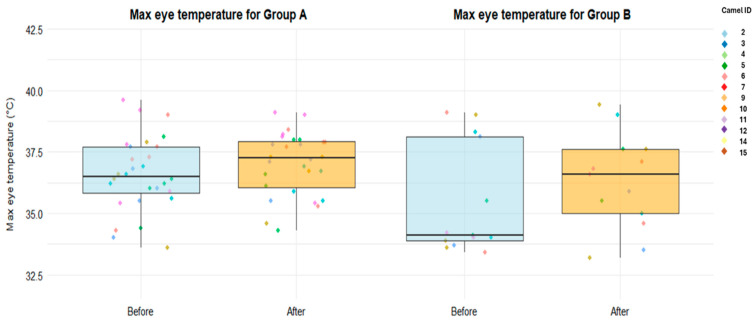
Boxplots of maximum eye temperature measured by infrared thermography (IRT) before and after training in group A and group B. Boxes represent the interquartile range, the central line indicates the median, and whiskers extend to the most extreme values within 1.5 times the interquartile range. Individual dots represent single observations, with colors corresponding to different camel IDs.

The overall mean maximum eye temperature was 36.5 °C (range: 33.2–39.6 °C). Mean values were 36.8 °C in camels from group A and 35.8 °C in those from group B. Before training, the mean maximum eye temperature was 36.22 °C (range: 33.4–39.6 °C), whereas after training it was 36.5 °C (range: 33.2–39.6 °C). Only the group had a significant effect on the maximum eye temperature (*p* = 0.019). Camels in group B showed a significantly lower maximum eye temperature compared with those in group A, being on average 0.92 °C lower (±0.40; *p* = 0.031). No significant effect of day or time was detected (*p* = 0.373 and *p* = 0.125, respectively). Nevertheless, a visual increase in maximum eye temperature after training was observed in group B (Figure 13), although this difference was not statistically significant.

## 4. Discussion

This study described how to train dromedary camels to self-load and unload using a positive reinforcement training method and evaluated its effects on behavior and welfare using infrared thermography. To the authors’ knowledge, this was the first study to apply this type of training to reach self-loading and unloading in dromedary camels, and it could be useful for educating camel industry members and stakeholders, particularly camel handlers and drivers worldwide.

Out of the twelve camels, eight successfully self-loaded and unloaded at least once during the training period, supporting our initial hypothesis. Previous studies have shown that positive reinforcement has been effective in training other livestock to self-load. For example, Ferguson et al. [15] showed that target training was effective in retraining sport horses that were considered difficult loaders, enabling them to self-load into a trailer. Another study showed that meat horses could be trained to self-load using target training, and this procedure helped reduce the negative effects of transportation [14]. The use of positive reinforcement to train camels should therefore be applied, as previously done with other animals, to facilitate easier and more efficient handling during transport procedures and to reduce stress levels during other husbandry procedures [31,32,33].

The camels included in the training showed slightly different behavioral responses upon temperament and approach tests. Particularly, the two groups (A and B) involved in the training had different experiences of human handling and interaction. However, during the training, both groups became easier to handle and improved their human–animal relationship due to the positive associations between the trainer, the ramp, the truck, and the feed [34]. Regardless of individual differences in the success of self-loading and unloading, all camels indeed learned to associate the clicker with the reward and to follow the trainer for a certain distance during the training period. The use of a clicker during training is helpful, provided that it is used properly, paired with a primary reinforcer, which motivates the animals [16]. On the first day, all camels except one successfully accepted the food reward, and thereafter, they all accepted it, becoming more familiar with it. Identifying the appropriate food reward and providing it as a primary reinforcer throughout the training is an important step in the success of animal training [35,36].

The number of clicks and the amount of reward tended to decrease slightly across training days, indicating that the camels learned to follow the trainer easily and learned to perform the desired activities more quickly. Positive reinforcement training, in particular, the clicker training used, has been demonstrated to be effective in training camels with different personalities and past handling experience. The literature reports that gender, physiological status, handler interaction, and herd size influence the cognitive ability of dromedary camels [37]. Such factors could be why we observed individual differences in the training outcomes. However, based on our findings, it seems crucial to adapt the training to each individual and evaluate progress day by day without overtraining the animal. Longer duration could indeed make the animal tired and reduce learning ability [38].

The average total training duration was one hour and twelve minutes over a maximum period of nine days, with an average daily training of 8.5 min per camel, and the fastest camel took only 30 min to learn to self-load. The loading and unloading of dromedary camels is usually chaotic and highly stressful for both camels and handlers. Usually, it takes hours, and a number of humans are needed to load a single camel into a vehicle [13]. A study by Fukasawa [11] stated that calf training improved loading efficacy and reduced stress on calves compared with non-trained ones. Dai et al. [14] reported that trained horses took less time compared to untrained horses. Yngvesson et al. [28] showed that the loading time of a horse at a vet clinic varied between 3 s and 112 min, while in sports events, loading varied between 3 s and 6.5 min. The loading time of horses significantly decreased after training, according to a study by Shanahan [39]. This is the first study to show that training dromedary camels to self-load and unload is possible and does not cause negative outcomes for animals and handlers, and it may be quicker than loading an untrained camel. Future studies should focus on the comparison between trained and untrained camels and the effects of training on their behavior and welfare during a transport event.

During the training, the phase of loading with the clicker decreased day by day, especially in the successful camels. Similarly, the time required to approach the truck showed a significant reduction across training days. Repeated exposure to non-aversive procedures or activities makes animals more accustomed to them [40,41]. This indicates that the training is progressing well and that the camels have learned to approach the truck, climb the ramp, and walk inside the truck with ease and minimal stress. Among all phases, the ramp took longer than the others, followed by approaching the truck. Specifically, unsuccessful camels spent a longer time on the ramp compared with successfully loaded camels. This result was expected, as loading is considered the most fearful and stressful phase of transport [42], and all the training to self-load focuses on reducing this innate fear. It is important to be very patient in this phase and adapt the training to the single behavioral response. With each additional training day, the odds of loading inside the truck increased by approximately 50%. The camels needed time to acclimate to the new stimuli of the ramp and the truck, which may have been intimidating. Habituation has been suggested to be important in camel training, helping them to accustom themselves to a new environment or stimulus when they are frequently exposed to it [27]. In our protocol, counterconditioning was applied using feed as positive reinforcement to allow the animal to shift from a negative association with the ramp and the truck (i.e., fear) to a positive association [34,43]. Future research should evaluate the difference between trained and untrained camels to understand the time difference and ease between the two groups and to better assess the applicability of this training method.

Overall, the duration of the training increased during the initial phase, peaked around the eighth day, and then declined. At the beginning of training, camels took longer to learn and become accustomed to the clicker phase and the ramp and truck. As the training days progressed, the training duration became shorter and shorter, showing that most camels achieved the training target by the eighth day and finished the daily training with shorter duration. Although there could be individual differences, this demonstrates that camels can be effectively trained in less than ten days. Studies have used different training durations for horses; for example, Shanahan [39] used a 30 min training session for two weeks. Other studies did not measure the training duration but defined success based on criteria such as self-loading three times a week [9]. We stopped training individual camels after they self-loaded three times in a row, but due to logistical reasons, we only trained for 10 days. Based on our results, the training protocol should last at least 8 consecutive days, but future studies should be conducted to determine the optimal training duration and test the camel’s ability to retain the learned behavior.

The number of forward steps on the ramp and in the truck increased over the training days, peaking on the ninth day, indicating that the camels took more forward steps as training progressed. Conversely, latency to take the first step on the ramp decreased over time, indicating a faster start to movement. Dai et al. [14], in their study, showed that trained horses made more forward steps toward the truck compared to untrained horses. This shows that the camels were learning and following instructions with minimal stress as the training day progressed. The observed frequencies of the other behavioral events were generally similar between groups. Successful camels showed a higher average occurrence of sniffing behavior toward the ramp or truck, while the unsuccessful camels showed more avoidance behavior. While there were no studies regarding the behavior of camels during loading and unloading, the sniffing behavior has been considered a key behavior that camels are keen to learn, and it is used to investigate whether a new object may pose any danger [44]. On the contrary, avoidance behavior could be a predictor of failure, as is usually shown in the case of fear of new stimuli [45]. It is therefore recommended to give time to the animals to sniff and explore the ramp and the truck and be more patient with those showing avoidance and other fear-related behavior.

IRT was used to assess possible stress and fear reactions caused by the training [21,46]. Camels in group B showed a lower maximum eye temperature compared to group A. This difference could be attributed to the fact that group A camels had minimal human interaction and handling experience, which may have caused them to experience slightly more stress than group B animals [47]. In addition, pregnant camels exhibit lower ocular temperature [22,48], and the presence of pregnant camels may have lowered the reading of the maximum eye temperature in group B camels. In general, there was no significant difference in maximum eye temperature before and after training, suggesting that the self-loading and unloading training did not cause stress or fear in the camels. Surprisingly, the camels in group A had a higher success rate (83.3%) than those in group B (50%), even if they were unbroken. This could be due to the different composition of the groups, as in group B, there were more pregnant females, which did not load, as they were probably more fearful and less keen to learn [37]. Other possible reasons could include differences in breeds, housing, and management. Future studies should confirm these preliminary findings and possibly suggest adapting the training depending on animal category, gender, and housing conditions.

Our findings should be interpreted with caution, as our study had several limitations. Firstly, there was the use of a handmade wooden ramp and a vehicle not typically designed to transport large animals such as dromedary camels. The ramp lacked protective side rails, and it had a slightly slippery surface; furthermore, the vehicle did not allow camels to turn inside easily. The use of properly designed vehicles and ramps could help achieve better and faster results, and it is strongly recommended [1,49]. The second limitation was the study field, such as machinery and other livestock, which sometimes distracted camels during the training. Moreover, the camels, except for the training hours, were not under our control, and their handling during the rest of the days and at night may have altered the training outcomes. Another limitation of the study was that we were unable to test the effectiveness of the training by comparing trained and untrained camels by transporting them to a certain location due to ethical constraints. Notwithstanding these limitations, this is the first study describing how to apply positive reinforcement training to teach camels to self-load and unload. Our manuscript could help improve camel welfare during transport.

## 5. Conclusions

Loading and unloading into vehicles is stressful and may endanger both camels and handlers. This study described how to train dromedary camels to self-load and unload step by step using a positive-reinforcement-based training. We proved that camels can learn to self-load into a truck without an increase in eye temperature measured using IRT, which may suggest the camels learned with minimal fear and distress. The daily training took about eight minutes; there were individual differences in the time required to learn and succeed. Overall, it is important that the training was conducted with patience, considering individual differences in behavioral responses and physiological states, and handling the camels accordingly. Our results should be confirmed by further studies that address the limitations reported above and evaluate the applicability and importance of this training for routine farm activities.

## Figures and Tables

**Table 1:** Ethogram for evaluation of camel behavior during the training.

Behavior	Measurement	Definition
Phases of the training		
Loading the clicker ^a^	Duration	The trainer provides feed and clicks, and the camel eats [16].
Approaching the truck ^a^	Duration	The camel walks from its station toward the ramp/truck following the trainer/target/container until reaching the ramp (modified from Ferguson and Rosales-Ruiz) [15].
Ramp ^a^	Duration	The camel stops in front of the ramp until it puts the first leg on the truck (modified from Ferguson and Rosales-Ruiz) [15].
Truck ^a^	Duration	The camel puts the first leg on the floor of the truck and steps, eats or stays inside the truck until it puts the first leg on the ramp to get down.
Unloading ^a^	Duration	The camel gets down from the truck, either facing forward or backward, moving its hind leg or front leg onto the ramp until it puts the first leg on the field [9].
Going back to the station	Duration	The camel walks back toward its station, and it follows the trainer/target at the end of the training session.
Behavioral states		
Lying ^b^	Duration	The animal is seated upright on its brisket with its legs tucked beneath the body, which is the natural resting posture of camels. The head may be lifted or resting on the ground [26].
Standing ^b^	Duration	The camel stands still on four legs [28].
Following ^b^	Duration	The camel does more than two complete steps and follows the trainer (modified from Aubè et al.) [26].
Feeding ^c^	Duration	The camel takes food into its mouth (plants or concentrate) and chews and swallows it after having been rewarded. The camel eats while walking and standing toward the ramp and back to its station/location [26].
Eating from the ramp ^c^	Duration	The camel ingests and eats feed from the ramp by himself without taking the feed from the trainer (modified from Aubè et al.) [26]
Eating from the truck ^c^	Duration	The camel ingests feed and chews and swallows from the truck by himself [9].
Behavioral events		
Clickers	Frequency	The trainer uses the clicker when the camel shows the desired behavior (e.g., going forward or touching the container) [16].
Food reward	Frequency	The camel is rewarded with food when it shows the desired behavior; it takes and eats concentrate/plants from the container or the hand of the trainer [16].
Steps forward on the ramp	Frequency	The camel lifts a leg and places it down on the ramp, moving forward to the trainer/container until it reaches the truck [14].
Steps backwards on the ramp	Frequency	The camel lifts a leg and places it down on the ramp, taking a backward step while it is on the ramp, moving away from the trainer/container [9].
Steps forward inside the truck	Frequency	The camel lifts a leg and places it on the floor of the truck, moving forward to the trainer/container
Steps backwards inside the truck	Frequency	The camel lifts a leg and places it down, taking a backward step while it is on the truck, moving away from the trainer/container
Stopping	Frequency	The camel stops or pauses from walking by itself [9].
Avoidance behavior	Frequency	The camel moves away from the trainer or the truck/ramp, moving its full body or part of it (i.e., its head, neck, front or back legs) sideways (modified from Yngvesson et al.) [28].
Sniffing the ramp/truck	Frequency	The camel explores the environment by bringing the nose into contact with an object (e.g., ramp or truck) [26].
Alert behavior	Frequency	The camel suddenly raises its head and extends its neck in response to a potential negative external stimulus (e.g., the sound of passing motorcycles/cars/tractors/machines)
Camel–trainer interaction	Frequency	The camel reaches the trainer with the head and interacts with them (sniffing, licking, touching, or rubbing) [27].
Sound emission	Frequency	The camel produces audible sounds from its mouth, such as loud groaning or moaning or grunting [26].
Defecation	Frequency	The camel drops feces [26].

Variables were measured as duration or frequency; behaviors indicated with the same superscript (i.e., a, b, c) are considered to be mutually exclusive.

**Table 2:** Results of the temperament, broken/unbroken test, approaching test, food choice, and the ability of the camels to accept food from the trainers on the first day of training.

Camel ID	Temperament Test	Approaching Test	Level of Tame	Food Reward/Treats	Reward/Food Acceptance from the Trainer
2	Aggressive	Negative	Unbroken	Concentrate	Yes
3	Curious	Negative	Unbroken	Plants	Yes
4	Friendly, shy	Neutral	Unbroken	Concentrate	Yes
5	Calm	Neutral	Unbroken	Plants and concentrate	Yes
6	Friendly, calm	Positive	Unbroken	Concentrate	Yes
7	Uneasy	Neutral	Unbroken	Plants and concentrate	Yes
9	Calm	Positive	Well-tamed	Concentrate	Yes
10	Fearful	Negative	Well-tamed	Did not accept food on the first day	No
11	Curious	Positive	tamed	Concentrate	Yes
12	Friendly, calm, shy	Positive	Tamed	Concentrate	Yes
14	Calm	Neutral	Tamed	Plants and concentrate	Yes
15	Shy	Neutral	Tamed	Plants and concentrate	Yes

**Table 3:** Summary of training outcomes, maximum distance on a ramp, total training duration until the camel loads for the first time, total training duration, and first day of successful loading in the truck.

Camel ID	Training Outcome	Maximum Distance on the Ramp (Meters)	Total Training Time to First Successful Loading (Seconds)	Total Training Durations (Seconds)	Day of the First Successful Loading
2	Successful and completed	3.5	2551	3805	7th
3	Successful and completed	3.5	2551	3563	5th
4	Non-successful	2	Not loaded	4108	
5	Successful: put his feet on the truck on the last day	3.5	4923	4923	10th
6	Successful and completed	3.5	4434	5903	8th
7	Successful/still to consider that he loaded twice	3.5	3899	4389	9th
9	Successful and completed	3.5	2457	4836	6th
10	Successful and completed	3.5	1812	3121	6th
11	Successful and completed	3.5	3764	4943	8th
12	Non-successful	3	Not loaded	4062	
14	Non-successful	2.8	Not loaded	3706	
15	Non-successful	2.8	Not loaded	4537	

**Table 4:** Descriptive statistics of the duration of each training phase measured in seconds stratified by training days.

Training Days	Training Phases (Time in Seconds)												Training Duration (Seconds)	
	Loading the Clicker		Approaching the Truck		Ramp		Truck		Unloading		Going Back to the Station			
	Mean ± SD	Min.–Max.	Mean ± SD	Min.–Max.	Mean ± SD	Min.–Max.	Mean ± SD	Min.–Max.	Mean ± SD	Min.–Max.	Mean ± SD	Min.–Max.	Mean ± SD	Min.–Max.
2	181.7 ± 92.8	82.6–418											181.7 ± 92.8	82.6–418
3	36.7± 41.3	1.8–149.6	181.0 ± 69.5	76.2–279.8							57.0 ± 19.2	29–91.2	274.8 ± 102.8	135.2–453.8
4	17.1 ± 17.675	0–61	130.2 ± 54.2	53.2–251.4	202.7 ± 104.3	0–389.4	0 ± 0	0–0			65.2 ± 24.8	34.8–100.4	415.2 ± 132.7	219–631.4
5	13.5 ± 17.76	0–48.2	101.5 ± 56.2	18.8–223.4	507.4 ± 346.7	0–1289	34.9 ± 77.9	0–197	51.3 ± 53.9	13.2–89.4	60.4 ± 52.3	20.2–198.4	718.4 ± 418.9	114.6–1485
6	13.0 ± 15.2	0–42.8	79.8 ± 90.9	8.6–341.8	363.4 ± 231.3	0–993.8	61.2± 129.6	0–418.2	54.5 ± 28.2	31–85.8	44.7 ± 17.5	17.4–76.4	570.7 ± 230.8	373.2–1169.2
7	15.9 ± 22.5	0–66.6	84.9 ± 96.6	12.2–336.2	453.4 ± 350.4	93.6–1350.2	67.3 ± 134.01	0–457.8	17.9 ± 13.9	4.6–36.8	45.2 ± 18.3	26.4–85.8	664.3 ± 321.6	327.8–1391
8	4.1 ± 6.6	0–19	38.6 ± 29.0	6.6–96.4	414.4 ± 239.6	60.2–825.8	82.9 ± 119.4	0–337.6	23.4 ± 17.4	5.4–49.6	44.3 ± 23.8	19.4–92.4	594.9 ± 163.5	296.2–879.4
9	2.6 ± 5.3	0–16	31.0 ± 24.9	3.8–72.2	426.0 ± 146.7	230.4–646.2	103.3 ± 161.8	0–385.8	16 ± 17.3	6–41.8	46.2 ± 52.6	1.8–175.4	616.4 ± 120.2	435.4–752.8
10	6.0 ± 9.3	0–22.6	20.7 ± 7.7	9–30	509.1 ± 347.6	120.4–1176	108.1 ± 166.4	0–453.8	9 ± 10.3	2.8–24.4	38.0 ± 30.8	15–99.4	675.0 ± 268.0	376.6–1226
Overall	35.5 ± 67.4	0–418	89.8 ± 79.5	3.8–341.8	407.8 ± 276.9	0–1350.2	63.5 ± 122	0–457.8	25.23 ± 24.9	2.8–89.4	50.8± 31.7	1.8–198.4	514.9 ± 289.8	82.6–1485

**Table 5:** Descriptive statistics of the steps (movement) made by the camels on the ramp and/or truck, stratified by training days.

Training Days	Behavioral Events (Frequency)									
	Step Forward on the Ramp		Step Backward on the Ramp		Step Forward Inside the Truck		Step Backward Inside the Truck		First step on the Ramp Latency Time	
	Mean ± SD	Min.–Max.	Mean ± SD	Min.–Max.	Mean ± SD	Min.–Max.	Mean ± SD	Min.–Max.	Mean ± SD	Min.–Max.
2										
3										
4	7.4 ± 7.6	0–26	0.4 ± 1.2	0–4	0 ± 0	0–0	0 ± 0	0–0	49.8 ± 43.1	1.6–120
5	29.3 ± 22.5	0–66	4.3 ± 3.2	0–10	0.6 ± 1.6	0–5	0.3 ± 0.9	0–3	27.9 ± 28.7	1.2–86.4
6	23.9 ± 15.3	0–46	2.7 ± 4.2	0–15	3.0 ± 5.4	0–15	0.7 ± 1.4	0–4	22.3 ± 23.0	2.2–68.4
7	32.8 ± 37.1	1–145	6.8 ± 9.1	0–29	4.92 ± 9.9	0–33	2.5 ± 4.2	0–11	21.7 ± 27.8	0.6–83.8
8	45.0 ± 22.6	11–96	11.9 ± 12.8	0–38	6.7 ± 8.3	0–22	2.2 ± 2.9	0–8	19.6 ± 20.7	3.2–70.4
9	46.4 ± 28.3	19–88	12.2 ± 12.6	0–39	6.1 ± 8.3	0–18	1.3 ± 2.2	0–6	8.8 ± 8.2	1.4–22.2
10	45.8 ± 19.4	24–83	9.8 ± 8.5	0–25	6.5 ± 8.	0–19	3.1 ± 4.6	0–11	7.5 ± 6.8	0.6–21.6
Overall	31.9 ± 26.2	0–145	6.5 ± 9.0	0–39	3.8 ± 7.2	0–33	1.4 ± 2.8	0–11	22.8 ± 27.4	0.6–120

## Data Availability

The data presented in this study are available from the corresponding author upon reasonable request.

## Supplementary Materials

The following supporting information can be downloaded at https://www.mdpi.com/article/10.3390/ani16071103/s1, Table S1: Age, sex, physiological state, coat colour, and breed of the dromedary camels involved in the study; Table S2: The nutritional composition of the concentrate used as feed reinforcer in the training; Table S3. The descriptive results obtained during the individual total days of training of loading the clicker, following some steps, touching the target, eating on the truck/ramp, putting one leg on the ramp, walking on the ramp, walking inside the truck, stratified by camel; Table S4: Summary of each camel activity per day, and the total training duration; Table S5: The descriptive results of loading the clicker, following some steps, touching the target, eating on the truck/ramp, putting one leg on the ramp, walking on the ramp, and walking inside the truck, stratified by training days.; Table S6: Descriptive statistics of the distance walked by camels during the full training period, distance walked on the ramp, and the number of attempts to walk onto the ramp by camels; Table S7: Descriptive results of the duration of the training phases stratified by camels; Table S8: Descriptive result of behavioural states of camels during the training stratified by camel; Table S9: Descriptive result of movement of camels on the ramp and/or truck stratified by camels; Table S10: Descriptive results of behavioural events recorded during the training stratified by camel; Table S11: Descriptive result of behavioural states of camels during the training stratified by training days; Table S12: Descriptive results of behavioural events recorded during the training stratified by training days; Table S13: Generalized linear mixed models (GLMMs) and a linear mixed-effects model (LMM) were used to evaluate the effects of day, training success, and their interaction on behavioral events and latency recorded during training, respectively. For the variables that resulted in significant data, the estimates are presented as ± standard error (SE) with 95% confidence intervals (CI). *p*-values for fixed effects, overall model *p*-values, and Akaike Information Criterion (AIC) are also reported; Table S14: Generalized linear mixed models (GLMMs) were used to evaluate the effects of day, training success, and their interaction on behavioural states recorded during training. For the variables that resulted in significant data, the estimates are presented as ± standard error (SE) with 95% confidence intervals (CI). *p*-values for fixed effects, overall model *p*-values, and Akaike Information Criterion (AIC) are also reported; Table S15: Linear mixed models (LMMs) evaluate the effects of day, training success, and their interaction on the duration of training phases. For the outcome variables, *Truck* and *Unloading*, only the effect of day was assessed. The variables that resulted in significant data are presented as estimates ± standard error (SE) with 95% confidence intervals (CI). *p*-values for fixed effects, overall model *p*-values, and Akaike Information Criterion (AIC) are also reported; Figure S1: Boxplots representing behavioral states duration (sec) stratified by training success (Yes/No). Boxes represent the interquartile range, the central line indicates the median, and whiskers extend to the most extreme values within 1.5 times the interquartile range. Individual dots represent single observations, with colors corresponding to different camel IDs; Figure S2: Boxplots representing behavioural events frequency (n/training duration) stratified by training success (Yes/No). Boxes represent the interquartile range, the central line indicates the median, and whiskers extend to the most extreme values within 1.5 times the interquartile range. Individual dots represent single observations, with colors corresponding to different camel IDs.

## References

[1] EFSA Panel on Animal Health and Welfare (AHAW) (2011). Scientific opinion concerning the welfare of animals during transport. EFSA J..

[2] Adams D. (1994). Transportation of animals and welfare. Rev. Sci. Tech.-Off. Int. Epizoot..

[3] FAO (2023). Livestock Transportation and Slaughter Practices–Practical Guidelines for Asia and the Pacific Region.

[4] Tarrant P. (1990). Transportation of cattle by road. Appl. Anim. Behav. Sci..

[5] Lambooij E. (2024). Animal Stress and Welfare During Transport and Slaughtering: An Outline for Future Policies. Animals.

[6] El Khasmi M. (2024). Stress transport in the dromedary camel. Dromedary Camel Behavior and Welfare: Camel Friendly Management Practices.

[7] Emeash H., Mostafa A., Karmy M., Khalil F., Elhussiny M.Z. (2016). Assessment of transportation stress in Dromedary camel (*Camelus dromedarius*) by using behavioural and physiological measures. J. Appl. Vet. Sci..

[8] Broom D.M. (2003). Causes of poor welfare in large animals during transport. Vet. Res. Commun..

[9] Dai F., Toson M., Bertotto D., Dalla Costa A., Heinzl E.U.L., Lega F., Minero M., Padalino B., Stefani A.L., Trestini S. (2025). Transportation to the Slaughterhouse: Can Training Reduce the Stress Response in Horses?. Vet. Sci..

[10] Bhatt N., Singh N.P., Mishra A.K., Kandpal D., Jamwal S. (2021). A detailed review of transportation stress in livestock and its management techniques. Int. J. Livest. Res..

[11] Fukasawa M. (2012). The calf training for loading onto vehicle at weaning. Anim. Sci. J..

[12] Nielsen S.S., Alvarez J., Bicout D.J., Calistri P., Canali E., Drewe J.A., Garin-Bastuji B., Gonzales Rojas J.L., Schmidt C.G., EFSA Panel on Animal Health and Welfare (AHAW) (2022). Welfare of cattle during transport. EFSA J..

[13] Helena Bauer J.H. The Welfare of Dromedary Camels During Road Transport in the Middle East. https://camelides.cirad.fr/fr/actualites/Docs/AA_Cameltransport%20in%20the%20Middle%20East_screen.pdf.

[14] Dai F., Dalla Costa A., Bonfanti L., Caucci C., Di Martino G., Lucarelli R., Padalino B., Minero M. (2019). Positive reinforcement-based training for self-loading of meat horses reduces loading time and stress-related behavior. Front. Vet. Sci..

[15] Ferguson D.L., Rosales-Ruiz J. (2001). Loading the problem loader: The effects of target training and shaping on trailer-loading behavior of horses. J. Appl. Behav. Anal..

[16] Feng L.C., Howell T.J., Bennett P.C. (2016). How clicker training works: Comparing reinforcing, marking, and bridging hypotheses. Appl. Anim. Behav. Sci..

[17] Hart B.L. (2011). The Art and Science of Clicker Training for Horses: A Positive Approach to Training Equines and Understanding Them.

[18] Cannas S., Palestrini C., Canali E., Cozzi B., Ferri N., Heinzl E., Minero M., Chincarini M., Vignola G., Dalla Costa E. (2018). Thermography as a non-invasive measure of stress and fear of humans in sheep. Animals.

[19] Stewart M. (2008). Non-Invasive Measurement of Stress and Pain in Cattle Using Infrared Thermography. Ph.D. Thesis.

[20] Redaelli V., Luzi F., Mazzola S., Bariffi G.D., Zappaterra M., Nanni Costa L., Padalino B. (2019). The use of infrared thermography (IRT) as stress indicator in horses trained for endurance: A pilot study. Animals.

[21] Arfuso F., Acri G., Piccione G., Sansotta C., Fazio F., Giudice E., Giannetto C. (2022). Eye surface infrared thermography usefulness as a noninvasive method of measuring stress response in sheep during shearing: Correlations with serum cortisol and rectal temperature values. Physiol. Behav..

[22] Faraz A., Masebo N.T., Hussain S.M., Waheed A., Ishaq H.M., Tauqir N.A., Abbasi A.R., Saleem F., Padalino B. (2025). Association of environmental temperature and relative humidity with ocular and flank temperatures in dromedary camels. Animals.

[23] Williams J., Friend T., Nevill C., Archer G. (2004). The efficacy of a secondary reinforcer (clicker) during acquisition and extinction of an operant task in horses. Appl. Anim. Behav. Sci..

[24] Menchetti L., Padalino B. (2024). Welfare assessment in dromedary camels. Dromedary Camel Behavior and Welfare: Camel Friendly Management Practices.

[25] Nielsen S.S., Alvarez J., Bicout D.J., Calistri P., Canali E., Drewe J.A., Garin-Bastuji B., Gonzales Rojas J.L., Schmidt C.G., EFSA Panel on Animal Health and Welfare (AHAW) (2022). Welfare of equidae during transport. EFSA J..

[26] Aubè L., Fatnassi M., Monaco D., Khorchani T., Lacalandra G.M., Hammadi M., Padalino B. (2017). Daily rhythms of behavioral and hormonal patterns in male dromedary camels housed in boxes. PeerJ.

[27] Le Meur C., Padalino B., Faye B. (2024). Camel handling and training. Dromedary Camel Behavior and Welfare: Camel Friendly Management Practices.

[28] Yngvesson J., de Boussard E., Larsson M., Lundberg A. (2016). Loading horses (*Equus caballus*) onto trailers—Behaviour of horses and horse owners during loading and habituating. Appl. Anim. Behav. Sci..

[29] Altmann J. (1974). Observational study of behaviour: Sampling methods. Behaviour.

[30] Smithson M., Verkuilen J. (2006). A better lemon squeezer? Maximum-likelihood regression with beta-distributed dependent variables. Psychol. Methods.

[31] Lomb J., Mauger A., Von Keyserlingk M., Weary D. (2021). Effects of positive reinforcement training for heifers on responses to a subcutaneous injection. J. Dairy Sci..

[32] Heinsius J., Lomb J., Lee J., von Keyserlingk M., Weary D. (2024). Training dairy heifers with positive reinforcement: Effects on anticipatory behavior. J. Dairy Sci..

[33] Laule G., Desmond T. (1998). Positive reinforcement training as an enrichment strategy. Second Nature: Environmental Enrichment for Captive Animals.

[34] Yorke A., Matusiewicz J., Padalino B. (2017). How to minimise the incidence of transport-related problem behaviours in horses: A review. J. Equine Sci..

[35] Martin A.L., Franklin A.N., Perlman J.E., Bloomsmith M.A. (2018). Systematic assessment of food item preference and reinforcer effectiveness: Enhancements in training laboratory-housed rhesus macaques. Behav. Process..

[36] Riemer S., Ellis S.L., Thompson H., Burman O.H. (2018). Reinforcer effectiveness in dogs—The influence of quantity and quality. Appl. Anim. Behav. Sci..

[37] Iglesias Pastrana C., Navas González F.J., Ciani E., McLean A.K., Delgado Bermejo J.V. (2025). Cognitive performance and variability in dromedary camels: Insights from a comparative psychometric approach. BMC Vet. Res..

[38] Bandini E., Tennie C. (2020). Exploring the role of individual learning in animal tool-use. PeerJ.

[39] Shanahan S. (2003). Trailer loading stress in horses: Behavioral and physiological effects of nonaversive training (TTEAM). J. Appl. Anim. Welf. Sci..

[40] Grandin T., Shivley C. (2015). How farm animals react and perceive stressful situations such as handling, restraint, and transport. Animals.

[41] Raderschall C.A., Magrath R.D., Hemmi J.M. (2011). Habituation under natural conditions: Model predators are distinguished by approach direction. J. Exp. Biol..

[42] Padalino B. (2015). Effects of the different transport phases on equine health status, behavior, and welfare: A review. J. Vet. Behav..

[43] Shnookal J., Tepper D., Howell T., Bennett P. (2024). Counterconditioning-based interventions for companion dog behavioural modification: A systematic review. Appl. Anim. Behav. Sci..

[44] Pastrana C.I., González F.J.N., Ciani E., McLean A.K., Bermejo J.V.D. (2024). Behavioural-type coping strategies in leisure dromedary camels: Factors determining reactive vs. proactive responses. Appl. Anim. Behav. Sci..

[45] Corgan M.E., Grandin T., Matlock S. (2021). Evaluating the reaction to a complex rotated object in the american quarter horse (*Equus caballus*). Animals.

[46] Stewart M., Webster J., Verkerk G., Schaefer A., Colyn J., Stafford K. (2007). Non-invasive measurement of stress in dairy cows using infrared thermography. Physiol. Behav..

[47] Fatnassi M., Padalino B. (2024). Behaviour: Behavioural repertoire and Behavioural needs of camels. Dromedary Camel Behavior and Welfare: Camel Friendly Management Practices.

[48] Abedellaah B., Sharshar A., Shoghy K., Rashed R. (2017). Normal ocular structure of dromedary camel (*Camelus dromedaries*): Gross, ultrasonographic and computed tomographic study. Assiut Vet. Med. J..

[49] Patil S.S., Bhangale J.H. (2025). Reducing Cattle Stress During Transport: Structural Design and Analysis of A Ramp-Gate System. Int. J. Environ. Sci..

