# Supplementary material for: How to Apply Positive-Reinforcement-Based Training for Self-Loading and Self-Unloading in Dromedary Camels
Source: Animals (Basel). 2026 Apr 3;16(7):1103. doi: 10.3390/ani16071103 (PMC13072205; doi:10.3390/ani16071103)
Supplement: Supplementary file 1 [file animals-16-01103-s001.zip › animals-4222285-supplementary.pdf]

Supplementary file one

Table S1: Age, sex, physiological state, coat colour, and breed of the dromedary camels involved in the study

Camel ID	Sex	Age (Years)	Physiological state	Coat colour	Breed
2	Male	5	Mature	White	Kohi
3	Male	5	Mature	White	Kohi
4	Male	4.5	Mature	White	Kohi
5	Male	5	Mature	White	Kohi
6	Male	5	Mature	White	Kohi
7	Male	4.5	Mature	White	Kohi
9	Female	7	Non-Pregnant	Brown	Barilya
10	Male	4	Mature	Brown	Barilya
11	Female	4	Non-Pregnant	Brown	Barilya
12	Female	4.5	Pregnant	Brown	Barilya
14	Female	4	Pregnant	Brown	Barilya
15	Female	4.5	Pregnant	Brown	Barilya

Table S2: The nutritional composition of the concentrate used as feed reinforcer in the training.

Note: All specifications are on dry matter (DM) basis

**Contents**	**Values**	**Measurement**
Dry Matter	~88	%min.
Moisture	12	%max.
Crude Protein	17-18	%
Crude Fat	4-7	%
Total Ash	12	%max.
Metabolizable Energy (ME)	~11.50	MJ/kg of DM
Net Energy for Lactation (NEL)	~7.00	MJ/kg of DM
Total Digestible Nutrients (TDN)	75	%max.
Acid Detergent Fiber (ADF)	10.75	%min.
Neutral Detergent Fiber (NDF)	24	%min.
**Additives as per 40 kg bag**		
Calcium	0.62	%
Phosphorus	0.99	%
Magnesium	0.53	%
Potassium	1.5	%
Sulfur	0.26	%
Sodium	0.42	%
Chlorine	0.68	%
Iron	43.98	Ppm
Zinc	46.22	Ppm
Copper	10.86	Ppm
Manganese	154.82	Ppm
Selenium	0.25	Ppm
Cobalt	4.54	Ppm
Iodine	0.03	Ppm

Table S3. The descriptive results obtained during the individual total days of training of loading the clicker, following some steps, touching the target, eating on the truck/ramp, putting one leg on the ramp, walking on the ramp, walking inside the truck, stratified by camel.

Camel ID	Load the clicker		Follow some steps		Touching target		Eating on the truck/ramp		Putting one leg on the ramp		Walking on the ramp		Walking inside the truck		Total number of days the camels trained
	Yes (%)	No (%)	Yes (%)	No (%)	Yes (%)	No (%)	Yes (%)	No (%)	Yes (%)	No (%)	Yes (%)	No (%)	Yes (%)	No (%)	
2	8 (100)	0 (0)	8 (100)	0 (0)	7 (87.5)	1 (12.5)	6 (85.7)	1 (14.3)	6 (100)	0 (0)	6 (100)	0 (0)	3 (50)	3 (50)	6
3	6 (100)	0 (0)	6 (100)	0 (0)	5 (83.3)	1 (16.7)	5 (100)	0 (0)	4 (100)	0 (0)	3 (75)	1(25)	3 (75)	1 (25)	9
4	9 (100)	0 (0)	9(100)	0 (0)	8 (88.9)	1 (11.1)	6 (75)	2 (25)	6 (85.7)	1 (14.3)	6 (85.7)	1(14.3)	0 (0)	7 (100)	9
5	9 (100)	0 (0)	9 (100)	0 (0)	8 (88.9)	1 (11.1)	6 (75)	2 (25)	6 (85.7)	1 (14.3)	6 (85.7)	1(14.3)	1 (14.3)	6 (85.7)	9
6	9 (100)	0 (0)	9(100)	0 (0)	8 (88.9)	1 (11.1)	8 (100)	0 (0)	7 (100)	0 (0)	6 (85.7)	1(14.3)	3 (42.9)	4 (57.1)	9
7	9 (100)	0 (0)	9 (100)	0 (0)	8 (88.9)	1 (11.1)	7 (87.5)	1 (12.5)	7 (100)	0 (0)	7 (100)	0(0)	2 (28.6)	5 (71.4)	9
9	7 (100)	0 (0)	7 (100)	0 (0)	7 (100)	0(0)	6 (100)	0 (0)	5 (100)	0 (0)	5 (100)	0(0)	3 (60)	2 (40)	7
10	7(10)	0(0)	7 (100)	0 (0)	6 (85.7)	1 (14.3)	6 (100)	0 (0)	5 (100)	0 (0)	4 (80)	1(20)	3 (60)	2 (40)	7
11	9(100)	0 (0)	9 (100)	0 (0)	9 (90)	0 (0)	8 (100)	0 (0)	7 (100)	0 (0)	7 (100)	0(0)	3 (42.9)	4 (57.1)	9
12	9 (100)	0 (0)	9(100)	0 (0)	8 (88.9)	1 (11.1)	4 (50)	4 (50)	5 (71.4)	2 (28.6)	5 (71.4)	2(28.6)	0 (0)	7 (100)	9
14	9 (100)	0 (0)	9(100)	0 (0)	8 (88.9)	1 (11.1)	7 (87.5)	1 (12.5)	7 (100)	0 (0)	7 (100)	0(0)	0 (0)	7 (100)	9
15	9 (100)	0 (0)	9(100)	0 (0)	8 (88.9)	1 (11.1)	7 (87.5)	1 (12.5)	6 (85.7)	1 (14.3)	6 (85.7)	1(14.3)	0 (0)	7 (100)	9

**Overall**	**100 (100)**	**0(0)**	**100 (100)**	**0 0**	**90 (90)**	**10 (10)**	**76 (86.4)**	**12 (13)**	**71 (93.4)**	**5 (6.6)**	**64 (88.9)**	**8 (11.1)**	**21 (27.6)**	**55 (72.4)**	

Table S4. Summary of each camel activity per day, and the total training duration.

Camel ID	Day 2 (Loading Clicker and Follow)	Day 3 (Truck)	Day 4 (Truck and Ramp)	Day 5 (Truck and Ramp)	Day 6 (Truck and Ramp)	Day 7 (Truck and Ramp)	Day 8 (Truck and Ramp)	Day 9 (Truck And Ramp)	Day 10 (Truck and Ramp)
2	LC, FS	LC, AT	LC, AT, L, ER	LC, AT, L	LC, AT, L	*LC, AT, L, ST, ET, UN, GB	*LC, AT, L, ST, ET, UN, GB	*LC, AT, L, ST, ET, UN, GB	Completed
3	LC, FS	LC, AT, ET	LC, AT, L, ER	*LC, AT, L, ST, ET, UN, GB	*LC, AT, L, ST, ET, UN, GB	*LC, AT, L, ST, ET, UN, GB	Completed	Completed	Completed
4	LC, FS	LC, AT	LC, AT, L	LC, AT, L, ER	LC, AT, L, ER	LC, AT, L	LC, AT, L	LC, AT, L	LC, AT, L
5	LC, FS	LC, AT	LC, AT, L	LC, AT, L, ER	LC, AT, L, ER	LC, AT, L	LC, AT, L	LC, AT, L	*LC, AT, L, ST, ET, UN, GB
6	LC, FS	LC, AT, ET	LC, AT, L, ER	LC, AT, L, ER	LC, AT, L, ER	LC, AT, L	*LC, AT, L, ST, ET, UN, GB	*LC, AT, L, ST, ET, UN, GB	*LC, AT, L, ST, ET, UN, GB
7	LC, FS	LC, AT, ET	LC, AT, L, ER	LC, AT, L,	LC, AT, L, ER	LC, AT, L, ER	LC, AT, L, ER	*LC, AT, L, ST, ET, UN, GB	*LC, AT, L, ST, ET, UN, GB
9	LC, FS	LC, AT, ET	LC, AT, L, ER	LC, AT, L, ER	*LC, AT, L, ST, ET, UN, GB,	*LC, AT, L, ST, ET, UN, GB,	*LC, AT, L, ST, ET, UN, GB	Completed	Completed
10	LC, FS	LC, AT, ET	LC, AT, L, ER	LC, AT, L	*LC, AT, L, ST, ET, UN, GB	*LC, AT, L, ST, ET, UN, GB	*LC, AT, L, ST, ET, UN, GB	Completed	Completed
11	LC, FS	LC, AT	LC, AT, L, ER	LC, AT, L	LC, AT, L, ER	LC, AT, L, ER	*LC, AT, L, ST, ET, UN, GB	*LC, AT, L, ST, ET, UN, GB	*LC, AT, L, ST, ET, UN, GB
12	LC, FS	LC, AT	LC, AT	LC, AT	LC, AT	LC, AT, L, ER	LC, AT, L, ER	LC, AT, L, ER	LC, AT, L, ER
14	LC, FS	LC, AT	LC, AT, L, ER	LC, AT, L, ER	LC, AT, L, ER	LC, AT, L, ER	LC, AT, L, ER	LC, AT, L, ER	LC, AT, L, ER
15	LC, FS	LC, AT	LC, AT	LC, AT, L	LC, AT, L, ER	LC, AT, L, ER	LC, AT, L, ER	LC, AT, L, ER	LC, AT, L, ER
Total animals trained	12	12	12	12	12	12	11	9	8

NB: *Show camels that successfully loaded and unloaded, LC: Loading the clicker, FS: Follow steps, AT: Approaching the truck, L: Loading, ER: Eating from the ramp, ST: Stay on the truck, ET: Eat from the truck, UL: Unloading, GB: Going back to the station

Table S5. The descriptive results of loading the clicker, following some steps, touching the target, eating on the truck/ramp, putting one leg on the ramp, walking on the ramp, and walking inside the truck, stratified by training days.

Training days	Number of camels trained each day	Load the clicker		Follow some steps		Touching target		Eating on the truck/ramp		Putting one leg on the ramp		Walking on the ramp		Walking inside the truck	
		Yes (%)	No (%)	Yes (%)	No (%)	Yes (%)	No (%)	Yes (%)	No (%)	Yes (%)	No (%)	Yes (%)	No (%)	Yes (%)	No (%)
2	12	12 (100)	0 (0)	12 (100)	0 (0)	2 (16.7)	10 (83.3)	NA	NA	NA	NA	NA	NA	NA	NA
3	12	12 (100)	0 (0)	12 (100)	0 (0)	12 (100)	0 (0)	7 (58.3)	5 (41.7)	NA	NA	NA	NA	NA	NA
4	12	12 (100)	0 (0)	12 (100)	0 (0)	12 (100)	0 (0)	8 (66.7)	4 (33.3)	8 (66.7)	4 (33.3)	5 (41.7)	7 (58.3)	0 (0)	12 (100)
5	12	12 (100)	0 (0)	12 (100)	0 (0)	12 (100)	0 (0)	11 (91.7)	1(8.3)	11 (91.7)	1(8.3)	11 (91.7)	1 (8.3)	2 (16.7)	10 (83.3)
6	12	12 (100)	0 (0)	12 (100)	0 (0)	12 (100)	0 (0)	11 (91.7)	1(8.3)	12 (100)	0 (0)	12 (100)	0 (0)	3 (25)	9 (75)
7	12	12 (100)	0 (0)	12 (100)	0 (0)	12 (100)	0 (0)	11 (91.7)	1(8.3)	12 (100)	0 (0)	12 (100)	0 (0)	4(33.3)	8 (66.7)
8	11	11 (100)	0 (0)	11 (100)	0 (0)	11 (100)	0 (0)	11 (100)	0 (0)	11 (100)	0 (0)	11 (100)	0 (0)	5(45.5)	6 (54.5)
9	9	9 (100)	0 (0)	9 (100)	0 (0)	9 (100)	0 (0)	9 (100)	0 (0)	9 (100)	0 (0)	9 (100)	0 (0)	4(44.4)	5 (55.6)
10	8	8 (100)	0 (0)	8 (100)	0 (0)	8 (100)	0 (0)	8 (100)	0 (0)	8 (100)	0 (0)	8 (100)	0 (0)	3(37.5)	5 (62.5)
Overall		100 (100)	0(0)	100(100)	0 (0)	90 (90)	10 (10)	76 (86.4)	12 (13)	71 (93.4)	5 (6.6)	64 (88.9)	8 (11.1)	21 (27.6)	55 (72.4)

Table S6. Descriptive statistics of the distance walked by camels during the full training period, distance walked on the ramp, and the number of attempts to walk onto the ramp by camels.

Camel ID	Distance walked by camels		Distance walked on the ramp		Number of attempts to walk onto the ramp	
	Mean ± SD	Min.-Max	Mean ± SD	Min.-Max	Mean ± SD	Min.-Max
2	30.38±11.74	3.5-42	2.53±1.34	0.5-3.5	1.67±1.21	1-4
3	32.5±16.83	2-52	2.63±1.75	0-3.5	1.25±0.5	1-2
4	37.89±15.77	3-60	1.26±0.75	0-2	2.43±1.4	1-5
5	36.72±23.62	3.5-80	1.46±1.17	0-3.5	1.43±0.79	1-3
6	40.5±19.18	2-70	2.7±1.3	0-3.5	1.14±0.38	1-2
7	48±28.58	3-100	2.24±1.44	0.2-3.5	1.14±0.38	1-2
9	32±12.71	10-49	3±0.87	1.5-3.5	1.4±0.89	1-3
10	27.71±8.62	10-36	2.6±1.52	0-3.5	1.2±0.45	1-2
11	30.5±11.49	10-49	2.71±1.04	1-3.5	4.14±1.77	2-7
12	33.67±11.85	15-55	1.49±1.27	0-3	2.17±1.47	0-4
14	27.78±6.91	15-40	1.73±0.84	0.5-2.8	2.71±1.38	1-5
15	33±14.35	15-65	1.71±0.88	0-2.8	2±0.82	1-3
Overall	34.53±16.6	2-100	2.12±1.24	0-3.5	1.95 ± 1.33	0-7

Table S7. Descriptive results of the duration of the training phases stratified by camels

Camel ID	Training phases (time in seconds)												Training duration (seconds)	
	Loading the clicker		Approaching		Ramp		Truck		Unloading		Going back to the station			
	Mean ± SD	Min-Max	Mean ± SD	Min-Max	Mean ± SD	Min-Max	Mean ± SD	Min-Max	Mean ± SD	Min-Max	Mean ± SD	Min-Max	Mean ± SD	Min-Max
2	51.63 ± 73.42	0-229.6	90.8 ± 42.98	42-153.4	300.77 ± 64.86	223.2-377.6	46.37 ± 53.35	0-112.6	8.53 ± 2.86	5.4-11	92.57 ± 40.52	53.4-175.4	475.63 ± 177.75	182.8-698
3	59.8 ± 66.71	2-187.8	143.72 ± 116.4	23.2-279.8	382.15 ± 236.16	249.6-736	96 ± 76.71	0-187.8	41.67 ± 43.39	4.6-89.4	89.76 ± 62.3	38.6-198.4	593.97 ± 388.78	187.8-1327
4	39.7 ± 63.19	0-193.2	82.91 ± 55.68	24.8-154.2	412.49 ± 152.28	165.8-594.4	0 ± 0	0-0			40.35 ± 18.01	21-75.8	456.47 ± 199.57	193.2-795.6
5	48.31 ± 73.21	1.8-239.2	129.35 ± 79.41	15.4-223.4	450.23 ± 376.27	93.6-1176	1.23 ± 3.25	0-8.6	2.8 ± NA	2.8-2.8	36.3 ± 15.17	17.4-59.6	547 ± 299.86	239.2-1226
6	15.49 ± 25.99	0-82.6	120.9 ± 96.45	26-336.2	450.51 ± 421.74	120.4-1289	158.6 ± 204.91	0-453.8	31.87 ± 24.28	4.2-49.6	54.65 ± 22.16	30.6-99.4	655.91 ± 439.53	82.6-1485
7	30.02 ± 39.94	0-118.8	72.38 ± 78.05	18.8-244	352.69 ± 127.89	185.8-496.6	80.74 ± 148.19	0-376.6	5.8 ± 1.7	4.6-7	61.85 ± 24.13	30.6-100.4	487.73 ± 171.06	118.8-718.2
9	82.66 ± 157.76	0-418	84.6 ± 76.28	15.2-228.8	307.68 ± 312.76	104.6-860.6	238.32 ± 195.81	0-457.8	37.1 ± 34.37	12.6-85.8	74.83 ± 18.07	41.4-93.8	630.51 ± 351.06	296.2-1248.8
10	36.86 ± 86.71	0-233.2	44.23 ± 46.69	6.6-122.6	220.56 ± 171.67	60.2-479.6	128.68 ± 139.62	0-337.6	28.2 ± 16.11	18.6-46.8	44.57 ± 11.6	27.8-59.4	374.51 ± 159.63	193.2-573.4
11	15.4 ± 39.31	0-119.4	31.77 ± 27.23	3.8-76.2	555.09 ± 354.2	363.2-1350.2	55.77 ± 81.65	0-213.8	20.47 ± 12.96	6-31	30.46 ± 13.38	8-44.4	549.27 ± 373.41	119.4-1391
12	16.46 ± 40.67	0-108.6	136.37 ± 129.71	8.6-341.8	483.93 ± 379.68	0-825.8	0 ± 0	0 ± 0			37.6 ± 14.88	19.4-60	507.83 ± 326.77	108.6-879.4
14	31.17 ± 53.4	0-150	59.91 ± 51.68	12.4-159	396.89 ± 154.16	152.8-594	0 ± 0	0 ± 0			32.38 ± 26.86	1.8-93.8	411.78 ± 167.42	150-651.8
15	14.71 ± 32.73	0-100.2	95.07 ± 82.24	6.4-246.8	483.51 ± 326.33	0-993.8	0 ± 0	0 ± 0			32.42 ± 14.98	15-54.8	504.11 ± 324.42	100.2-1169.2

Overall	35.48 ± 67.37	0-418	89.77 ± 79.54	3.8-341.8	407.84 ± 276.99	0-1350.2	63.49±122	0-457.8	25.23 ± 24.94	2.8-89.4	50.77 ± 31.7	1.8-198.4	514.92 ± 289.81	82.6-1485

Table S8. Descriptive result of behavioural states of camels during the training stratified by camel

Camel ID	Behavioural states (time in seconds)											
	**Lying**		**Standing**		**Following**		**Feeding**		**Eating from the ramp**		**Eating from the truck**	
	Mean ± SD	Min-Max	Mean ± SD	Min-Max	Mean ± SD	Min-Max	Mean ± SD	Min-Max	Mean ± SD	Min-Max	Mean ± SD	Min-Max
2	1.52 ± 4.31	0-12.2	406.28 ± 167.93	134.8-586	67.15 ± 22.12	47-112	395.52 ± 134.07	181.6-563.6	28.33 ± 69.4	0-170	0 ± 0	0-0
3	15.37 ± 20.98	0-50.8	504.6 ± 375.53	138.8-1225.8	73.3 ± 24.31	41.6-101.2	454.37 ± 283.06	181.4-988.8	51.2 ± 81.89	0-171.8	45.88 ± 55.66	0-124
4	3.29 ± 9.87	0-29.6	398.58 ± 200.5	136.4-717	54.51 ± 12.28	33.8-71.8	352.78 ± 144.4	193.2-648.4	33.49 ± 30.84	0-88.2	0 ± 0	0-0
5	7 ± 11.59	0-31.6	478.58 ± 313.82	163-1195.8	61.11 ± 15.65	30.2-79.2	418.58 ± 229.53	169.2-949.4	34.86 ± 34.48	0-85	2.94 ± 7.79	0-20.6
6	2.11 ± 6.33	0-19	597.4 ± 435.14	44.8-1419	56.16 ± 14.98	35.6-75.2	493.47 ± 326.67	79.6-1138.2	11.94 ± 29.7	0-79.2	78.06 ± 123.82	0-308.2
7	7.27 ± 19.12	0-57.8	409.51 ± 171.2	80-646.8	70.76 ± 24.93	38-116	262.07 ± 104.99	100.2-430	69.03 ± 92.1	0-232.2	53.83 ± 85.01	0-221.6
9	0 ± 0	0-0	571.94 ± 342.3	256-1141.2	58.46 ± 29.5	26.2-107.6	510.29 ± 273.09	285.6-967	20.04 ± 35.98	0-83	18.93 ± 31.07	0-73
10	0 ± 0	0-0	327.8 ± 168.52	132-528	46.4 ± 18	19-71.2	274.83 ± 150.83	134.4-512.2	6.68 ± 14.94	0-33.4	57.43 ± 89.63	0-231.4
11	0 ± 0	0-0	508.16 ± 378.83	81.4-1361.6	41.11 ± 22.38	9.2-77.6	394.69 ± 252.94	111-971.6	41.8 ± 71.31	0-189.6	0 ± 0	0-0
12	0 ± 0	0-0	456.28 ± 340.56	22.4-844.2	51.55 ± 23.67	14.8-92.2	387.08 ± 227.69	106.8-708	17.87 ± 23.89	0-60.6	0 ± 0	0-0
14	0 ± 0	0-0	371.31 ± 172.08	122.2-598.4	40.44 ± 23.56	9.8-80.6	316.4 ± 116.89	123.4-475.6	14.29 ± 14.99	0-41.2	0 ± 0	0-0
15	0 ± 0	0-0	461.24 ± 324.34	48.6-1101.4	42.53 ± 19.18	12.8-68.4	392.09 ± 270.16	93.8-1002	4.37 ± 7.65	0-18.2	0 ± 0	0-0
Overall	2.84 ± 9.65	0-57.8	457.06 ± 289.95	22.4-1419	54.78 ± 22.65	9.2-116	385.39 ± 219.88	79.6-1138.2	27.79 ± 49.37	0-232.2	22.17 ± 59.03	0-308.2

Table S9. Descriptive results of the movement of camels on the ramp and/or truck stratified by camels

Camel ID	Behavioural events (frequency)									
	Step forward on the ramp		Step backward on the ramp		First step on the ramp latency time		Step forward inside the truck		Step backward inside the truck	
	Mean ± SD	Min-Max	Mean ± SD	Min-Max	Mean± SD	Min-Max	Mean ± SD	Min-Max	Mean ± SD	Min-Max
2	36.17±27.8	5-86	2.5 ± 2.88	0-7	22.1 ± 23.63	5.4-69	3.5 ± 4.72	0-12	0.83 ± 1.33	0-3
3	33.25 ± 20.55	4-49	1.25 ± 2.5	0-5	36.4 ± 55.82	5.2-120	4.5 ± 3.11	0-7	1.5 ± 1.73	0-3
4	25.71 ± 16.46	5-56	10.86 ± 9.17	0-24	13.63 ± 8.7	2.2-28	0 ± 0	0-0	0 ± 0	0-0
5	17.43 ± 29.66	0-83	3.43 ± 4.12	0-12	50.5 ± 31.74	5.8-83.8	0.14 ± 0.38	0-1	0 ± 0	0-0
6	30.71 ± 16.84	2-55	1.14 ± 1.57	0-4	17.6 ± 29.06	0.6-81.6	7.86 ± 9.81	0-19	3.14 ± 4.56	0-11
7	17.43 ± 7.74	7-26	2.86 ± 3.24	0-8	10.54 ± 11.63	1.6-31.6	5 ± 8.54	0-18	1.57 ± 2.7	0-6
9	34.8 ± 15.96	11-56	2 ± 4.47	0-10	11.32 ± 5.69	3.2-16.4	11.4 ± 13.09	0-33	3.4 ± 4.1	0-10
10	22.4 ± 10.36	4-28	2 ± 2.83	0-6	16.16 ± 13.04	5.2-38.2	10.4 ± 9.91	0-22	4 ± 4.53	0-11
11	74.43 ± 39.85	26-145	16.29 ± 14.96	0-39	21.39 ± 37.34	0.6-98.8	5.86 ± 7.45	0-16	2.86 ± 3.76	0-9
12	31.83 ± 26.38	0-62	7.83 ± 7.25	0-17	16.85 ± 13.4	3.6-34.2	0 ± 0	0-0	0 ± 0	0-0
14	32.57 ± 22.45	11-75	8.86 ± 7.88	0-20	23.83 ± 24.62	4.8-68.4	0 ± 0	0-0	0 ± 0	0-0
15	25.43 ± 20.03	0-53	13.86 ± 13.97	0-38	36.77 ± 35.85	5-86.4	0 ± 0	0-0	0 ± 0	0-0
Overall	31.91 ± 26.19	0-145	6.51 ± 9.01	0-39	22.75 ± 27.43	0.6-120	3.89 ± 7.19	0-33	1.4 ± 2.84	0-11

Table S10. Descriptive results of behavioural events recorded during the training stratified by camel

Camel ID	Behavioural events (frequency)																	
	Clicker		Reward		Stopping		Avoidance behaviour		Sniffing the ramp/truck		Alert behaviour		Camel trainer interaction		Sound emission		Defecation	
	Mean ± SD	Min-Max	Mean ± SD	Min-Max	Mean ± SD	Min-Max	Mean ± SD	Min-Max	Mean ± SD	Min-Max	Mean ± SD	Min-Max	Mean ± SD	Min-Max	Mean ± SD	Min-Max	Mean ± SD	Min-Max
2	35.63 ± 14.16	14-49	23.88 ± 7.88	11-30	1.13 ± 1.13	0-3	0.88 ± 0.83	0-2	0.17 ± 0.41	0-1	1.88 ± 1.55	0-5	0.13 ± 0.35	0-1	0 ± 0	0-0	0 ± 0	0-0
3	37.5 ± 19.93	14-73	24.67 ± 17.01	14-58	0.67 ± 0.82	0-2	0.67 ± 0.82	0-2	1 ± 1.15	0-2	0.33 ± 0.52	0-1	0.17 ± 0.41	0-1	1.5 ± 1.64	0-4	0 ± 0	0-0
4	25 ± 11.58	9-44	16.78 ± 5.67	10-26	0.67 ± 0.71	0-2	2 ± 2	0-6	0.29 ± 0.49	0-1	0.67 ± 1	0-3	0.11 ± 0.33	0-1	3 ± 2.96	1-9	0 ± 0	0-0
5	36.33 ± 25.76	12-95	25.33 ± 16.06	10-62	0.22 ± 0.67	0-2	0.89 ± 0.78	0-2	0.57 ± 1.13	0-3	0.78 ± 1.64	0-5	0.11 ± 0.33	0-1	3 ± 3.24	0-10	0.11 ± 0.33	0-1
6	49.67 ± 30.98	6-94	30.67 ± 18.36	7-58	1.44 ± 1.88	0-6	0.67 ± 0.87	0-2	1 ± 1.41	0-3	1 ± 1.12	0-3	0.11 ± 0.33	0-1	0.22 ± 0.44	0-1	0 ± 0	0-0
7	23.78 ± 10.73	9-43	14.22 ± 5.93	8-22	0.33 ± 0.5	0-1	1.44 ± 1.74	0-5	2.14 ± 2.61	0-7	0.22 ± 0.67	0-2	0.11 ± 0.33	0-1	1 ± 1.22	0-3	0 ± 0	0-0
9	46 ± 34.23	13-100	25.29 ± 11.9	14-44	0 ± 0	0-0	0.57 ± 0.79	0-2	1.8 ± 2.49	0-6	0.29 ± 0.49	0-1	0 ± 0	0-0	0.14 ± 0.38	0-1	0.29 ± 0.49	0-1
10	25.57 ± 12.23	14-42	16 ± 5.51	11-27	0 ± 0	0-0	0.71 ± 1.5	0-4	2 ± 1.87	0-5	0.57 ± 0.79	0-2	0 ± 0	0-0	0 ± 0	0-0	0 ± 0	0-0
11	35.44 ± 29.61	8-106	22.67 ± 14.49	10-58	0 ± 0	0-0	2 ± 1.87	0-5	4.57 ± 4.58	0-13	1 ± 0.87	0-2	0.22 ± 0.44	0-1	0 ± 0	0-0	0.67 ± 1.12	0-3

12	28.13 ± 20.23	6-62	21.25 ± 12.42	6-43	0 ± 0	0-0	1.75 ± 1.91	0-5	1.67 ± 1.63	0-4	0.38 ± 0.74	0-2	0 ± 0	0-0	0.38 ± 0.52	0-1	0 ± 0	0-0
14	26.67 ± 14.34	8-57	18.44 ± 7.23	9-30	0 ± 0	0-0	1.44 ± 1.33	0-4	1.14 ± 2.27	0-6	0.33 ± 0.71	0-2	0.11 ± 0.33	0-1	0 ± 0	0-0	0 ± 0	0-0
15	20.78 ± 16.12	3-55	15.56 ± 9.79	3-37	0 ± 0	0-0	0.89 ± 1.05	0-3	3.86 ± 3.39	0-9	0.44 ± 0.73	0-2	0 ± 0	0-0	0.11 ± 0.33	0-1	0 ± 0	0-0
Overall	32.27 ± 22.01	3-106	21.12 ± 12.18	3-62	0.37 ± 0.86	0-6	1.19 ± 1.41	0-6	1.72 ± 2.55	0-13	0.67 ± 1.03	0-5	0.09 ± 0.29	0-1	0.8 ± 1.77	0-10	0.09 ± 0.41	0-3

Table S11. Descriptive result of behavioural states of camels during the training stratified by training days

Training days	Behavioural stats (time in seconds)											
	Lying		Standing		Following		Feeding		Eating from the ramp		Eating from the truck	
	Mean ± SD	Min.-Max.	Mean ± SD	Min.-Max.	Mean ± SD	Min.-Max.	Mean ± SD	Min.-Max.	Mean ± SD	Min.-Max.	Mean ± SD	Min.-Max.
2	0 ± 0	0-0	129.4 ± 83.06	44.8-353.8	51.63 ± 16.29	27.8-74.4	167.22 ± 78.93	79.6-344.6				
3	4.82 ± 16.69	0-57.8	199.22 ± 88.46	81.4-391	70.35 ± 18.98	47-116	233.87 ± 64.76	134.4-321.4			99.2 ± 40.2	57.8-142
4	4.62 ± 15.32	0-50.8	338.87 ± 114.78	173.2-506	71.64 ± 15.13	45.8-94.8	305.2 ± 74.21	180.2-438.2	81.44 ± 89.81	0-232.2	0 ± 0	0-0
5	7.7 ± 12.33	0-31.6	648.13 ± 410.3	22.4-1419	62.53 ± 24.1	38-107.6	552.77 ± 317.64	113.6-1138.2	24.6 ± 36.56	0-88.2	7.65 ± 25.39	0-84.2
6	0.93 ± 3.23	0-11.2	513.63 ± 229.26	308.8-1101.4	55.7 ± 15.42	33.8-81.2	484.5 ± 208.4	271.8-1002	10.1 ± 12.65	0-33	1.93 ± 6.39	0-21.2
7	4.12 ± 9.9	0-30.2	612.7 ± 325.46	254.6-1361.6	47.17 ± 15.78	26.2-75.2	492.83 ± 250.4	169.2-971.6	8.32 ± 23	0-78.8	8 ± 18.95	0-55.4
8	0 ± 0	0-0	546.42 ± 157.57	265.2-844.2	48.6 ± 20.12	19-81	420.84 ± 146.07	178-664.2	25.2 ± 49.2	0-166.8	25.04 ± 69.7	0-231.4
9	1.36 ± 4.07	0-12.2	569.56 ± 104.18	411.4-701.6	45.42 ± 33.9	9.2-112	394.53 ± 115.41	187.8-563.6	30.42 ± 31.87	0-85	46.2 ± 91.93	0-221.6
10	0.95 ± 2.69	0-7.6	643.5 ± 274.71	323-1195.8	30.37 ± 19.23	11.2-62.8	430.25 ± 247.99	184.8-949.4	15.13 ± 22.76	0-65	49.48 ± 107.12	0-308.2
Over all	2.84 ± 9.65	0-57.8	457.06 ± 289.95	22.4-1419	54.78 ± 22.65	9.2-116	385.39 ± 219.88	79.6-1138.2	27.79 ± 49.37	0-232.2	22.17 ± 59.03	0-308.2

Table S12. Descriptive results of behavioural events recorded during the training stratified by training days

Training days	Behavioural events (frequency)																	
	Clicker		Reward		Stopping		Avoidance behaviour		Sniffing the ramp/truck		Alert behaviour		Camel trainer interaction		Sound emission		Defecation	
	Mean ± SD	Min.-Max.	Mean ± SD	Min.-Max.	Mean ± SD	Min.-Max.	Mean ± SD	Min.-Max.	Mean ± SD	Min.-Max.	Mean ± SD	Min.-Max.	Mean ± SD	Min.-Max.	Mean ± SD	Min.-Max.	Mean ± SD	Min.-Max.
2	11.33 ± 4.85	3-20	11.33 ± 4.44	3-20	0 ± 0	0-0	0.17 ± 0.58	0-2			0 ± 0	0-0	0 ± 0	0-0	0.5 ± 1.17	0-4	0 ± 0	0-0
3	19 ± 6.34	10-31	17.25 ± 5.28	10-27	0.33 ± 0.78	0-2	1.08 ± 0.67	0-2			0.75 ± 0.97	0-2	0.08 ± 0.29	0-1	0.83 ± 1.4	0-4	0 ± 0	0-0
4	26.45 ± 11.34	11-47	15.45 ± 4.13	10-24	0.55 ± 0.82	0-2	1.36 ± 1.63	0-5	0.18 ± 0.6	0-2	1.27 ± 1.42	0-5	0 ± 0	0-0	0.55 ± 0.82	0-2	0 ± 0	0-0
5	46.33 ± 29.94	6-100	29.5 ± 16.26	6-58	0.42 ± 0.67	0-2	1.75 ± 1.36	0-4	2.17 ± 2.72	0-7	0.75 ± 0.97	0-3	0.08 ± 0.29	0-1	0.92 ± 1.31	0-3	0.08 ± 0.29	0-1
6	35.83 ± 18.03	17-78	20.5 ± 9.05	8-37	0.33 ± 0.49	0-1	1 ± 1.13	0-3	1.33 ± 2.1	0-6	0.5 ± 0.67	0-2	0 ± 0	0-0	0.92 ± 2.57	0-9	0 ± 0	0-0
7	41.58 ± 29.84	9-106	25.08 ± 17.24	9-58	0.5 ± 1.73	0-6	1.42 ± 1.56	0-5	2.25 ± 3.91	0-13	0.58 ± 1	0-3	0.08 ± 0.29	0-1	1.17 ± 2.82	0-10	0.25 ± 0.45	0-1
8	42.18 ± 16.8	15-75	23.73 ± 9.84	9-43	0.36 ± 0.67	0-2	2.27 ± 2.1	0-6	1.27 ± 1.49	0-4	0.55 ± 0.82	0-2	0.18 ± 0.4	0-1	1.09 ± 2.47	0-7	0.18 ± 0.6	0-2
9	33.89 ± 17.39	8-63	23.89 ± 10.93	8-40	0.67 ± 1.12	0-3	0.78 ± 1.39	0-4	2 ± 2.5	0-6	1.44 ± 1.67	0-5	0.44 ± 0.53	0-1	0.78 ± 1.3	0-4	0 ± 0	0-0
10	35.75 ± 25.4	17-95	25.13 ± 16.3	10-62	0.25 ± 0.46	0-1	0.75 ± 0.89	0-2	3.25 ± 2.71	1-9	0.25 ± 0.46	0-1	0 ± 0	0-0	0.25 ± 0.71	0-2	0.38 ± 1.06	0-3
Overall	32.27 ± 22.01	3-106	21.12 ± 12.18	3-62	0.37 ± 0.86	0-6	1.19 ± 1.41	0-6	1.72 ± 2.55	0-13	0.67 ± 1.03	0-5	0.09 ± 0.29	0-1	0.8 ± 1.77	0-10	0.09 ± 0.41	0-3

Table S13. Generalized linear mixed models (GLMMs) and a linear mixed-effects model (LMM) were used to evaluate the effects of day, training success, and their interaction on behavioral events and latency recorded during training, respectively. For the variables that resulted in significant data, the estimates are presented as ± standard error (SE) with 95% confidence intervals (CI). P-values for fixed effects, overall model p-values, and Akaike Information Criterion (AIC) are also reported.

**Variable**	**Estimate ± SE**	**95% CI**	**p-value**	**Model LRT p-value**	**AIC**
Behavioural events (GLMMs)					
Loading step forward on the ramp				< 0.001	607.8627
Day	0.26 ± 0.03	(0.19, 0.32)	< 0.001		
Training success			0.572		
Loading step backward on the ramp				< 0.001	396.2712
Day	0.56 ± 0.16	(0.26, 0.87)	< 0.001		
Training success			0.104		
Day * Training success			0.018		
- No	Ref.				
- Yes	- 0.46 ± 0.20	(- 0.85, - 0.08)	0.018		
Clicker				0.022	705.8569
Day	- 0.02 ± 0.01	(-0.04, 0.00)	0.098		
Training success			0.020		
- No	Ref.				
- Yes	0.20 ± 0.09	(0.03, 0.38)	0.020		
Reward				< 0.001	645.8192
Day	- 0.06 ± 0.01	(- 0.09, - 0.04)	< 0 .001		
Training success			0.574		
Avoidance behaviour				0.016	285.5647
Day			0.961		
Group			0.466		
Day * Training success			0.066		
- No	Ref.				
- Yes	- 0.18 ± 0.10	(- 0.37, 0.01)	0.066		
Sniffing ramp/truck				0.093	253.628
Day	0.18 ± 0.08	(0.02, 0.35)	0.032		
Training success			0.925		
Latency time (LMM)					
First step on the ramp				< 0.001	233.4693
Day	− 0.28 ± 0.07	(- 0.42, - 0.14)	< 0.001		
Training success			0.156		

P-values in bold refer to the statistical significance or trend towards the significance of the predictive variable in the model; the significance of a category against the reference is reported in regular font
Estimates are reported on the log scale.
Training success = 'No' was used as the reference category; interaction terms represent deviations from the reference level.
Ref: reference category.

Table S14. Generalized linear mixed models (GLMMs) were used to evaluate the effects of day, training success, and their interaction on behavioural states recorded during training. For the variables that resulted in significant data, the estimates are presented as ± standard error (SE) with 95% confidence intervals (CI). P-values for fixed effects, overall model p-values, and Akaike Information Criterion (AIC) are also reported.

**Variable**	**Estimate ± SE**	**95% CI**	**p-value**	**Model LRT p-value**	AIC
Behavioural states					
Feeding				< 0.001	-154.9869
Day	- 0.31 ± 0.05	(- 0.40, - 0.22)	< 0.001		
Training success			0.009		
– No	Ref.				
– Yes	- 1.16 ± 0.45	(- 2.05, - 0.29)	0.009		
Day * Training success			0.033		
– No	Ref.				
– Yes	0.13 ± 0.06	(0.01, 0.24)	0.033		
Standing				< 0.001	-234.7215
Day	0.26 ± 0.02	(2.08, 0.31)	< 0.001		
Training success			0.435		
Following				< 0.001	-249.1947
Day	- 0.26 ± 0.02	(- 0.31, - 0.21)	< 0.001		
Training success			0.250		

P-values in bold refer to the statistical significance or trend towards the significance of the predictive variable in the model; the significance of a category against the reference is reported in regular font

Estimates are reported on the logit scale.

Training success = 'No' was used as the reference category; interaction terms represent deviations from the reference level.

Ref: reference category.

Table S15. Linear mixed models (LMMs) evaluate the effects of day, training success, and their interaction on the duration of training phases. For the outcome variables, *Truck* and *Unloading*, only the effect of day was assessed. The variables that resulted in significant data are presented as estimates ± standard error (SE) with 95% confidence intervals (CI). P-values for fixed effects, overall model p-values, and Akaike Information Criterion (AIC) are also reported.

**Variable**	**Estimate ± SE**	**95% CI**	**p-value**	**Model LRT p-value**	AIC
Phases of the training					
Loading the clicker				< 0.001	233.39
Day	- 0.35 ± 0.06	(- 0.47, - 0.22)	< 0.001		
Training success			0.561		
Approaching				< 0.001	190.3972
Day	- 0.34 ± 0.03	(- 0.40, - 0.28)	< 0.001		
Training success			0.721		
Loading the ramp				0.060	150.4781
Day			0.436		
Training success			0.038		
– No	Ref.				
– Yes	- 0.37 ± 0.18	(- 0.73, - 0.03)			
Truck					60.39783
Day			0.741		
Unloading					65.61571
Day	- 0.38 ± 0.12	(- 0.61, - 0.15)	0.001		

P-values in bold refer to the statistical significance or trend towards the significance of the predictive variable in the model; the significance of a category against the reference is reported in regular font
Estimates are reported on the log scale.
Training success = 'No' was used as the reference category; interaction terms represent deviations from the reference level.
Ref: reference category.

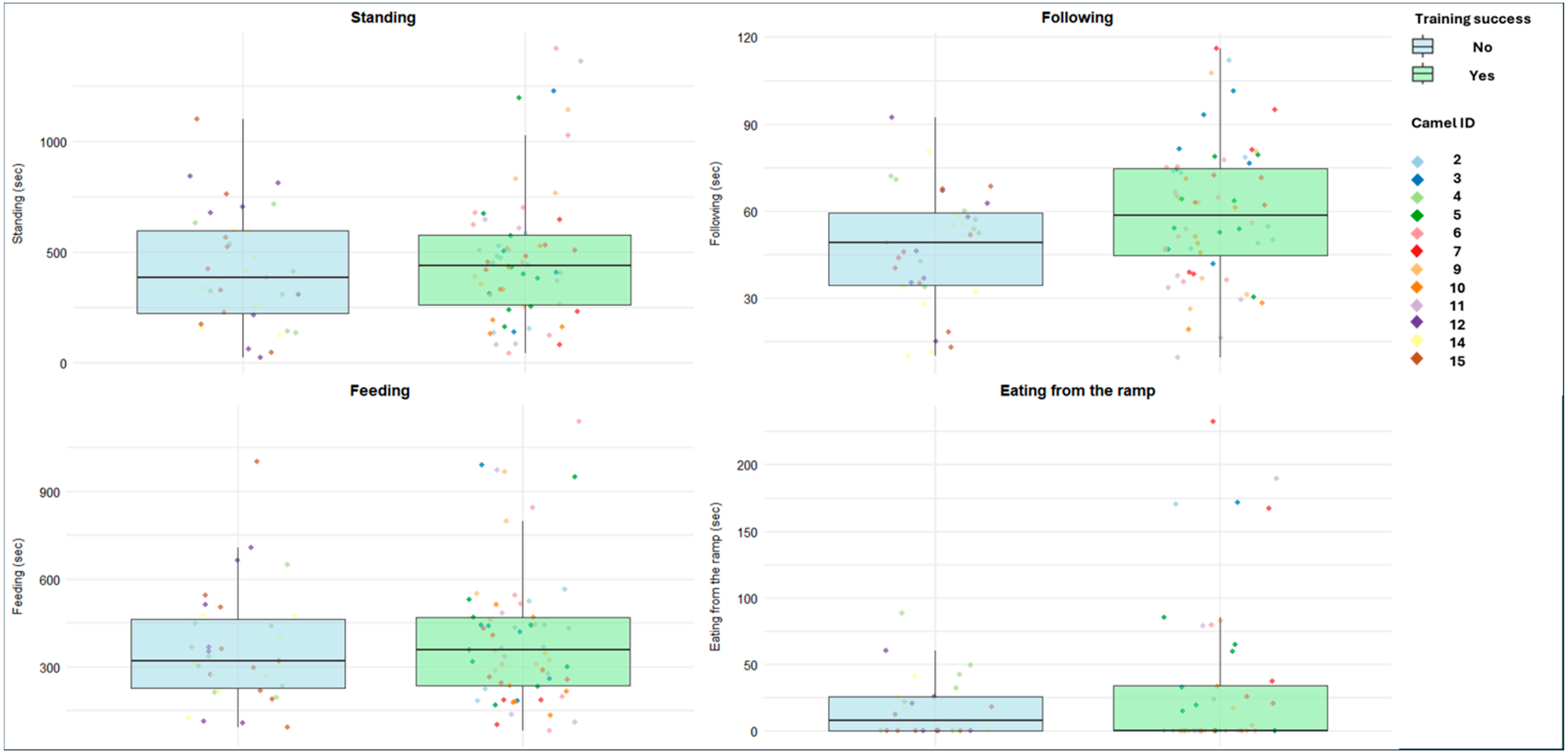

Figure S1. Boxplots representing behavioral states duration (sec) stratified by training success (Yes/No). Boxes represent the interquartile range, the central line indicates the median, and whiskers extend to the most extreme values within 1.5 times the interquartile range. Individual dots represent single observations, with colors corresponding to different camel IDs

.

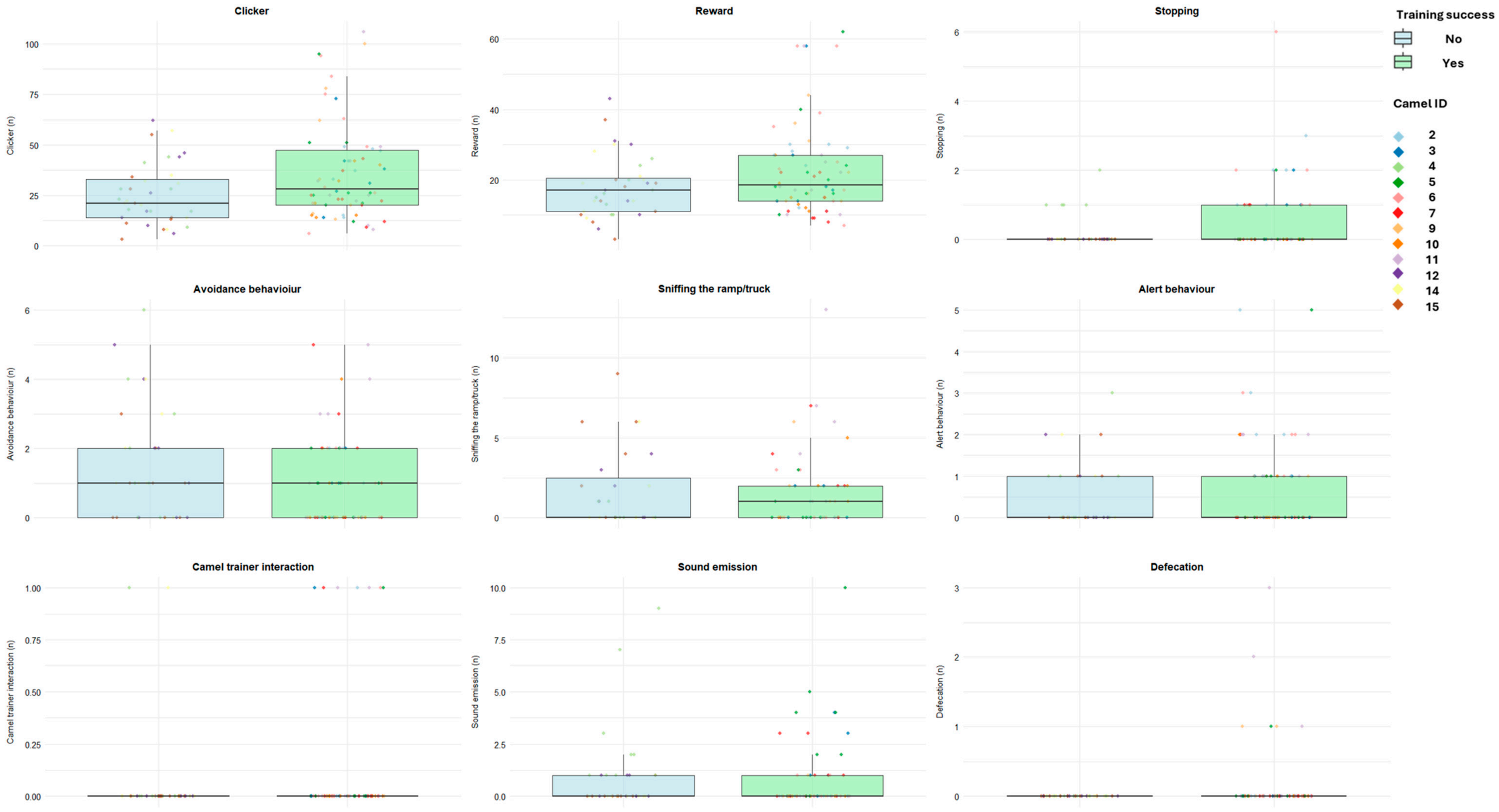

Clicker
Reward
Stopping
Avoidance behavioiur
Sniffing the ramp/truck
Alert behaviour
Camel trainer interaction
Sound emission
Defecation
Clicker (n)
Reward (n)
Stopping (n)
Avoidance behaviour (n)
Sniffing the ramp/truck (n)
Alert behaviour (n)
Camel trainer interaction (n)
Sound emission (n)
Defecation (n)
Training success
No
Yes
Camel ID
2
3
4
5
6
7
9
10
11
12
14
15

Figure S2. Boxplots representing behavioural events frequency (n/training duration) stratified by training success (Yes/No). Boxes represent the interquartile range, the central line indicates the median, and whiskers extend to the most extreme values within 1.5 times the interquartile range. Individual dots represent single observations, with colors corresponding to different camel IDs.
